# Adaptive Evolution of the *Myo6* Gene in Old World Fruit Bats (Family: Pteropodidae)

**DOI:** 10.1371/journal.pone.0062307

**Published:** 2013-04-19

**Authors:** Bin Shen, Xiuqun Han, Gareth Jones, Stephen J. Rossiter, Shuyi Zhang

**Affiliations:** 1 Institute of Molecular Ecology and Evolution, Institutes for Advanced Interdisciplinary Research, East China Normal University, Shanghai, China; 2 School of Biological Sciences, University of Bristol, Bristol, United Kingdom; 3 School of Biological and Chemical Sciences, Queen Mary University of London, London, United Kingdom; University of Western Ontario, Canada

## Abstract

Myosin VI (encoded by the *Myo6* gene) is highly expressed in the inner and outer hair cells of the ear, retina, and polarized epithelial cells such as kidney proximal tubule cells and intestinal enterocytes. The *Myo6* gene is thought to be involved in a wide range of physiological functions such as hearing, vision, and clathrin-mediated endocytosis. Bats (Chiroptera) represent one of the most fascinating mammal groups for molecular evolutionary studies of the *Myo6* gene. A diversity of specialized adaptations occur among different bat lineages, such as echolocation and associated high-frequency hearing in laryngeal echolocating bats, large eyes and a strong dependence on vision in Old World fruit bats (Pteropodidae), and specialized high-carbohydrate but low-nitrogen diets in both Old World and New World fruit bats (Phyllostomidae). To investigate what role(s) the *Myo6* gene might fulfill in bats, we sequenced the coding region of the *Myo6* gene in 15 bat species and used molecular evolutionary analyses to detect evidence of positive selection in different bat lineages. We also conducted real-time PCR assays to explore the expression levels of *Myo6* in a range of tissues from three representative bat species. Molecular evolutionary analyses revealed that the *Myo6* gene, which was widely considered as a hearing gene, has undergone adaptive evolution in the Old World fruit bats which lack laryngeal echolocation and associated high-frequency hearing. Real-time PCR showed the highest expression level of the *Myo6* gene in the kidney among ten tissues examined in three bat species, indicating an important role for this gene in kidney function. We suggest that *Myo6* has undergone adaptive evolution in Old World fruit bats in relation to receptor-mediated endocytosis for the preservation of protein and essential nutrients.

## Introduction

Myosin VI (encoded by the *Myo6* gene), a member of the actin filament-based molecular motor proteins, is the only myosin known to move towards the minus end of the actin filament thus far [1] and appears to be involved in a wide range of cellular functions such as clathrin-mediated endocytosis, cell migration, vesicular membrane traffic, cell migration and mitosis [2], [3].

*Myo6* is expressed in the actin-rich cuticular plate of inner and outer hair cells of the ear and is fundamental for the development and maintenance of stereocilia [2], [4], [5]. At least three mutations in *Myo6* have been associated with non-syndromic deafness in humans probably because of disruptions to the structure and function of stereocilia [6], [7]. Bats use echolocation, usually involving ultrasonic frequencies for orientation and often for foraging [8]. Echolocating bats perhaps have the most sensitive high-frequency hearing among mammals, and echolocation calls emitted by most echolocating bats range in dominant frequency from 11 kHz to over 200 kHz [9]. Such excellent auditory performance making echolocating bats fascinating mammals for studying genes associated with hearing. Recently, many studies have revealed that some genes associated with hearing have undergone positive selection in echolocating bats and cetaceans [10], [11], [12], [13], [14], [15], [16]. Considering the important role of *Myo6* in hearing, it is reasonable to hypothesize that the *Myo6* may also be a target gene for positive selection in bats that use laryngeal echolocation compared with species that do not use laryngeal echolocation (the Old World fruit bats in the family Pteropodidae).

*Myo6* is also expressed abundantly in the photoreceptor cells and retinal pigment epithelial (RPE) cells in the retina [17], [18]. Moreover, evidence from myosin VI functional null *Snell’s waltzer* (*sv*/*sv*) mice showed that myosin VI contributes to the normal functioning of retinal electrophysiology [17]. Thus myosin VI also plays an unknown but important role in vision. It was suggested that laryngeal echolocating bats use echolocation rather than vision as major means of perceiving their environment [19], [20], [21]. However, in addition to using olfaction [22], pteropodids without the ability of laryngeal echolocation presumably rely more on vision for orientation and finding food than other laryngeal echolocating bats. Thus it is also reasonable to hypothesize that positive selection may act on *Myo6* in pteropodids, as species in this lineage use vision primarily for orientation.

Moreover, myosin VI is highly expressed in polarized epithelial cells such as kidney proximal tubule cells and intestinal enterocytes, where it is associated with clathrin-mediated endocytosis [23], [24], [25]. In the kidney, large amounts of glomerular-filtered serum proteins are reabsorbed by proximal tubule cells relying on a process called receptor (megalin/cubulin)-mediated endocytosis [26]. A recent study revealed that myosin VI plays an important role in this process via vesicle formation and the transportation of vesicles towards early endosomes [27]. Renal proximal tubule reabsorption is very important for health, being responsible for the clearance of the vast majority of proteins filtered by the glomerulus. The impairment of this process will cause proteinuria, an excess of serum proteins in the urine [26]. More importantly, as many serum proteins are carrier proteins binding many essential components including vitamins and trace elements, receptor-mediated endocytosis also accounts for the preservation of many essential serum components such as vitamin D, vitamin B_12_ and iron [26], [28], [29], [30], [31].

Among the bats (Chiroptera), Old World fruit bats and New World fruit bats (Phyllostomidae) have independently evolved a high-carbohydrate but low-nitrogen diet comprising mainly fruit and/or nectar [32]. Although some New World fruit bats are known to supplement their diet with insects [33], [34] and Old World fruit bats also accidentally or even deliberately consume insects [35], [36], recent studies with stable-isotope analyses showed that Old World fruit bats and New World fruit bats in the subfamily Stenodermatinae are predominantly frugivorous [32], [34]. Most fruits that frugivorous bats mainly eat contain only 0.2–1.4% dry weight protein [37]. Nectar also contains only traces of amino acids [38]. Besides, fruits are also lacking many essential vitamins, especially fat-soluble vitamins such as vitamin D and vitamin B_12_ [39]. Captive pteropodids fed on fruit-only diets show nutrient deficiencies and even neurological impairment caused by vitamin B_12_ deficiency [40], [41]. Hence, how Old World fruit bats subsist on diets with low contents of protein and essential nutrients has long been of interest, and earlier studies focusing on behavior and physiology revealed some clues [42], [43], [44]. Because *Myo6* is involved in the critical importance of receptor-mediated endocytosis [27] for the preservation of protein and essential nutrients such as vitamins [26], we hypothesized that the *Myo6* gene may have undergone positive selection in the Old World fruit bats and/or the New World fruit bats.

In this study, we sequenced the coding region of the *Myo6* gene from 15 bat species, and studied the molecular evolution of this gene in bats and other mammals. We also used real-time PCR assay to determine patterns of gene expression in a range of organs, including the eye, cochlea and kidneys, to understand where in the body proteins with potential functional significance are expressed. With the combination of molecular evolutionary analyses and real-time PCR assay, we intend to test our hypotheses that *Myo6* has undergone positive selection in echolocating bats for their high-frequency hearing, in Old World fruit bats for their effective vision and/or in Old World fruit bats and New World fruit bats for the preservation of protein and essential nutrients.

## Materials and Methods

### Ethics Statement

Our procedures involving animals were in accordance with the guidelines of Regulations for the Administration of Laboratory Animals (Decree No. 2 of the State Science and Technology Commission of the People’s Republic of China on November 14, 1988). Bats were sacrificed by cervical dislocation and their tissues were sampled immediately. Protocols were approved by the Animal Ethics Committee of East China Normal University (ID No: 20101002). The neotropical bat species were sampled in Mexico during April, 2010 for our previous study [45] with assistance from Professor Rodrigo A. Medellín and Dr. Rafael Avila-Flores of the Instituto de Ecología, Universidad Nacional Autónoma de México, under the scientific collecting license number FAUT-0250, issued in the name of their collaborator, Dr. Gerardo Suzán.

### Taxonomic Coverage

We sequenced the coding region of the *Myo6* gene from 15 bat species covering eight of the 17 extant chiropteran families. From the suborder Yinpterochiroptera, we included three Old World fruit bats from the family Pteropodidae that do not use laryngeal echolocation (*Cynopterus sphinx*, *Rousettus leschenaultii* and *Eonycteris spelaea*). Also from the Yinpterochiroptera we sampled five insectivorous echolocating bats from sister families to the Old World fruit bats: *Megaderma lyra* (Megadermatidae), *Rhinolophus ferrumequinum* and *R. pusillus* (Rhinolophidae) and *Hipposideros pratti* and *H. armiger* (Hipposideridae). For echolocating bats from the suborder Yangochiroptera, we studied two New World fruit bats *Artibeus lituratus* and *Leptonycteris yerbabuenae* (Phyllostomidae) and five insectivorous bats representing three families: *Tadarida plicata* (Molossidae), *Myotis ricketti* and *Pipistrellus abramus* (Vespertilionidae) and *Mormoops megalophylla* and *Pteronotus parnellii* (Mormoopidae). The family Mormoopidae is sister to the New World fruit bats. All new *Myo6* sequences were deposited to GenBank and accession numbers are JX023444-JX023458.

We also obtained available published *Myo6* sequences of nine other mammal species from GenBank to provide a greater phylogenetic coverage for molecular evolutionary analyses: *Homo sapiens* (NM_004999), *Pan troglodytes* (XM_001144940), *Mus musculus* (NM_001039546), *Rattus norvegicus* (XM_001061392), *Bos taurus* (NM_001206072), *Canis familiaris* (XM_862495), *Ailuropoda melanoleuca* (XM_002923922), *Equus caballus* (XM_001503608) and *Sus scrofa* (NM_214021). Details on echolocation and food habits for all bat species and their corresponding *Myo6* accession numbers are listed in Table S1.

### Isolation, Amplification and Sequencing

For these 15 bat species, total RNA was isolated from brain tissue (stored at −80°C) using Trizol reagent (Invitrogen) following the standard protocol, and 5 ug total RNA was reverse-transcribed into cDNA by SuperScript™ III Reverse Transcriptase kit (Invitrogen). The coding sequence of the *Myo6* gene was divided into four overlapping fragments (∼1000 bp for each) (Figure S1), and four pairs of primers were designed to amplify these four fragments (Table S2). All PCR products were isolated using a 1% agarose gel and purified with a Gel Extraction Kit (Qiagen), then ligated into pGEM-T easy vector (Promega), cloned and sequenced using the Terminator kits (Applied Biosystems) on an ABI 3730 DNA sequencer. Then the sequences of four fragments were assembled together to obtain the full length of *Myo6* coding sequences. Considering the specific expression of *Myo6* isoforms in brain and polarized epithelial cells such as kidney proximal tubule cells reported in a previous study [25], we cloned the fourth fragment of *Myo6* coding sequences from kidney cDNA for six bat species: *C*. *sphinx* and *R*. *leschenaultii* for the Old World fruit bats, *R*. *ferrumequinum* and *H*. *armiger* as representatives of yinpterochiropteran laryngeal echolocating bats and *M*. *ricketti* and *Scotophilus kuhlii* for yangochiropteran laryngeal echolocating bats. *S. kuhlii* (a close relative of *P. abramus* also from the family Vespertilionidae) was used because we did not have kidney tissue from *P. abramus*. Comparisons of isoforms from brain and kidney tissues showed that the only differences between kidney and brain isoforms was that isoform expressed in the brain contained a 9aa small insertion and those from kidney contained a 32aa large insertion (Figure S1), which was congruent with the results from a previous study [25].

### Phylogenetic Reconstruction

The nucleotide sequences of 24 species were aligned using ClustalX [46] and coding sequences were translated to amino acids using MEGA4 [47]. Then the Bayesian phylogenetic tree was reconstructed based on the aligned nucleotide sequences using MrBayes 3.1.2 [48] with the TIM3+I+Γ nucleotide substitution model selected by jModelTest0.1 [49]. For the Bayesian analysis, we performed 10,000,000 generations of MCMC and sampled every 100 generations, with the first 2,000,000 generations discarded as burn-in, since the standard deviations of split frequencies were stable below 0.01 after 2,000,000 generations of MCMC performances. All other options and priors were the default settings of MrBayes 3.1.2 software.

### Molecular Evolution Analyses

Since the potential occurrence of recombination could adversely affect the power and accuracy of phylogenetic reconstruction [50] and the detection of positive selection [51], we first detected whether there was evidence of recombination in our dataset before molecular evolutionary analyses. We conducted GARD [52] in the HyPhy package [53] to detect the evidence of statistically supported recombination breakpoints (thus recombination).

A species topology with 24 mammals was constructed based on the accepted species relationships [54], [55], [56]. To test for positive selection in *Myo6*, we estimated the rate of nonsynonymous substitutions (d_N_) and synonymous substitutions (d_S_) using PAML CODEML [57].

We first performed two-ratio models, in which the d_N_/d_S_ ratios (termed as omega or ω) was allowed to differ between the foreground and the background. Firstly, to test selection pressure of *Myo6* gene in echolocating bats, separate two-ratio models were conducted with the foreground branch set as the ancestral branch leading to Chiroptera (all bats), Yinpterochiroptera, Yinpterochiroptera echolocating bats and Yangochiroptera, respectively. We also conducted two-ratio models on the ancestral branch leading to Old World constant frequency (CF) rhinolophids (species from the families of Hipposideridae and Rhinolophidae), because species of this lineage could emit CF echolocation calls at high-duty-cycles [58] and strong evidence of positive selection has recently been found acting on a key hearing gene (*Prestin*) in this lineage [10], [14], [16]. Finally, we also conducted two-ratio models on the ancestral branches leading to the Old World fruit bats and the New World fruit bats, respectively. In all cases, the one-ratio model, which assumes the equal *d*_N_/*d*_S_ ratio among all branches, was performed as a null model.

We also applied the test 2 of branch-site model A to detect positively selected sites along particular branches [59]. In this model, the phylogeny is separated into two portions: the fixed branches are set as foreground and the remaining branches as background. Four site classes of codons are assumed (class 0, class 1, class 2a and class 2b). Class 0 and class 1 codons are assumed to evolve under purifying selection (0<ω_0_<1) and neutral selection (ω_1_ = 1) respectively throughout the phylogeny. Class 2a and class 2b evolve under, respectively, purifying and neutral selection on the background, but are grouped together as class 2 and allowed to evolve under positive selection (ω_2_>1) on the foreground. The null model was the modified branch-site model A with the ω_2_ fixed as 1. We applied the test 2 of branch-site model A to the above seven ancestral branches which were tested by two-ratio models, i.e., ancestral branches leading to Chiroptera, Yinpterochiroptera, Yinpterochiroptera echolocating bats, Yangochiroptera, Old World CF rhinolophids, Old World fruit bats and New World fruit bats. All the results of alternative and null hypotheses were compared using likelihood ratio tests (LRTs).

In addition to the methods of PAML, two alternative approaches were used to detect the evidence of positive selection in our dataset. First, the random effects branch-site model (Branch-site REL) [60] was applied in the HyPhy package [53] to examine site-wise variation across branches. Since no uniform section pressure was enforced across all background branches assumed to be not under positive selection, this method might be more robust to errors. Second, using the Datamonkey web server (http://www.datamonkey.org/), we conducted the GA-Branch method [61], in which a range of ω classes were assigned to branches without *a priori* designation of focal lineages of interest.

### Real-time PCR

Real-time PCR assays were performed to determine the expression of the *Myo6* gene in major bat tissues. Three representative bat species were used: *C*. *sphinx* (a frugivorous bat from the Old World fruit bats), *R*. *ferrumequinum* (an insectivorous yinpterochiropteran echolocating bat) and *M*. *ricketti* (an insectivorous yangochiropteran echolocating bat). Adult individuals of these three bat species were sampled from the wild in China. Individuals were sacrificed humanely after capture and brain, eye, cochlea, heart, lung, liver, stomach, intestine, kidney and pectoral muscle tissues were stored in liquid nitrogen immediately for RNA preservation. For each species, three adult individuals were used for replication. Total RNA was isolated using Trizol reagent (Invitrogen) following the protocol, and cDNA was synthesized from 5 ug total RNA using SuperScript™ III Reverse Transcriptase kit (Invitrogen). Gene expression was analyzed using SYBR®Premix Ex Taq™ (TaKaRa) in the ABI PRISM 7300 real-time PCR system (Applied Biosystems) following the protocol (See Table S2 for primer information). The amount of cDNA template for each tissue was fixed to 100 ng. The PCR products were sequenced for confirmation. The amount of *Myo6* were normalized to the *Gapdh* gene [62], [63] and arbitrarily calibrated to pectoral muscle for each species using the 2^−ΔΔCt^ method [64]. The Kruskal-wallis test was used to test for significant differences of the *Myo6* mRNA expression levels within ten tissues for each species. And pairwise comparison was done by the two-tailed nonparametric Mann-Whitney U test, when the Kruskal-wallis test yielded a statistically significant value (*P*<0.05). *P*-value <0.05 was considered as significant.

## Results

Our final *Myo6* gene sequence dataset comprised 24 taxa, including three Old World fruit bats (Pteropodidae), two frugivorous New World fruit bats (Phyllostomidae) and other ten insectivorous laryngeal echolocating bats. The alignment of *Myo6* coding sequences comprised 3861 nucleotides, equating to 1287 amino acids, of which 169 amino acids (∼13%) were variable in eutherian mammals (Figure S2).

**Figure 1:**
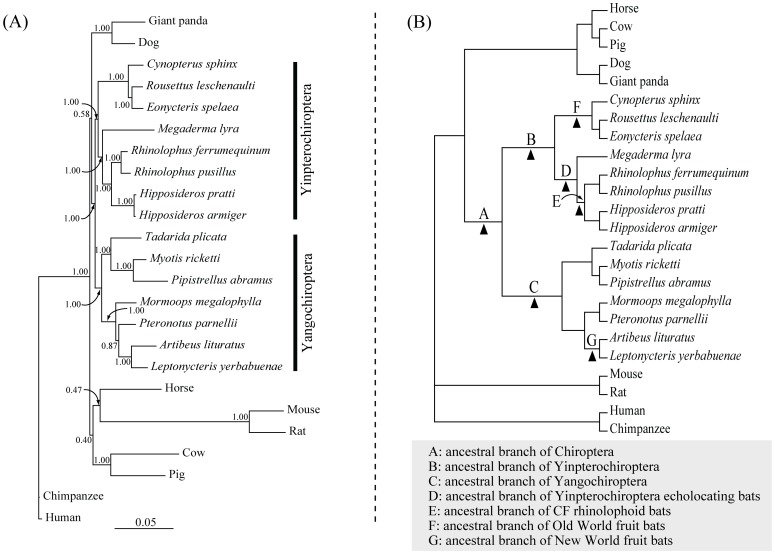
Unconstrained Bayesian phylogenetic tree and species topology. (A) Unconstrained Bayesian phylogenetic tree based on *Myo6* gene coding sequences, under the model of TIM3+I+Γ. Values on the nodes are posterior probabilities. (B) The species tree of 24 mammals based on accepted bat species relationships (see Materials and Methods). Seven branches tested both by the two-ratio model tests and the branch-site model A tests were marked by A, B, C, D, E, F and G, respectively.

Our Bayesian phylogenetic reconstruction based on the coding sequences of *Myo6* revealed a tree in which the major groupings agreed with the accepted mammal species tree (Figure 1). Hence the species of Pteropodidae (*C. sphinx*, *R. leschenaultii* and *E. spelaea*) grouped with species in the family Rhinolophidae (*R. ferrumequinum* and *R*. *pusillus*), Hipposideridae (*H. pratti* and *H*. *armiger*) and Megadermatidae (*M. lyra*) to comprise the clade Yinpterochiroptera [Bayesian posterior probability (BPP) = 1.00]. Other bats (*T. plicata*, *M. megalophylla*, *P. parnellii*, *M. ricketti*, *P. abramus*, *A. lituratus* and *L. yerbabuenae*) grouped together and comprised the clade Yangochiroptera (BPP = 1.00) (Figure 1A). We found no evidence of recombination breakpoints (i.e. recombination) in our dataset and hence excluded any potentially adverse influences of recombination in our phylogenetic reconstructions and subsequent molecular evolutionary analyses.

In order to detect selection pressures acting on the *Myo6* gene in echolocating bats, Old World fruit bats and New World fruit bats, we conducted a number of two-ratio model tests to seven branches (branch A∼G in Figure 1B). Results of all two-ratio model tests are shown in Table 1. Our results of two-ratio models which set the foreground as the ancestral branch leading to Chiroptera, Yinpterochiroptera, Yangochiroptera, Yinpterochiroptera echolocating bats and Old World constant frequency (CF) rhinolophids (branches marked with A, B, C, D and E in Figure 1B), respectively, showed no elevated ω values on these ancestral branches compared with the ω values of the corresponding background branches (Table 1). These results indicated that no selective pressure change of *Myo6* was occurred in those major focal branches leading to echolocating bats. The two-ratio model test for New World fruit bats (the branch marked with G in Figure 1B) exhibited similar results, with the two-ratio model which set the ancestral branch leading to New World fruit bats as the foreground showing no significantly better fit than the null (one-ratio) model [likelihood ratio test (LRT) statistic (2Δ*ℓ*) = 0.012, df = 1, *P*>0.05] (Table 1). However, the two-ratio model which designed the ancestral branch of Old World fruit bats (the branch marked with F in Figure 1B) as foreground was a significantly better fit to the dataset than the one-ratio model (2Δ*ℓ* = 19.611, df = 1, *P*<0.001) (Table 1). The estimated ω value on the ancestral branch of Old World fruit bats was an order of magnitude greater than that of background (0.135 versus 0.037, respectively, Table 1), indicating a selection pressure change acting on *Myo6* in the Old World fruit bats.

**Table 1:** Results of two-ratio model tests of selection pressure on the *Myo6* gene in bats.

Model	np	*ℓ*	ω_0_ a	ω_Fix_ a	Model Compared	2Δ*ℓ*	*P*
A. One ratio: ω_0_	47	−15988.40	0.039	= ω_0_			
B. Two ratios: ω_0_, ω_A_	48	−15987.25	0.039	0.0001	B vs. A	2.293	>0.05
C. Two ratios: ω_0,_ ω_B_	48	−15987.58	0.039	0.0001	C vs. A	1.644	>0.05
D. Two ratios: ω_0,_ ω_C_	48	−15986.36	0.039	0.0001	D vs. A	4.072	0.044
E. Two ratios: ω_0,_ ω_D_	48	−15988.40	0.039	0.036	E vs. A	0.005	>0.05
F. Two ratios: ω_0,_ ω_E_	48	−15988.33	0.039	0.0497	F vs. A	0.138	>0.05
G. Two ratios: ω_0,_ ω_F_	48	−15978.59	0.037	**0.135**	G vs. A	19.611	<0.001
H. Two ratios: ω_0,_ ω_G_	48	−15988.39	0.039	0.037	H vs. A	0.012	>0.05

a ω_Fix_ (ω_A_, ω_B_, ω_C_, ω_D_, ω_E_, ω_F_ and ω_G_) and ω_0_, are the ω ratios for branches A, B, C, D, E, F, G and other branches, respectively (see Figure 1B).

Then we performed the test 2 of branch-site model A to detect the positively selected sites on the above seven ancestral branches leading to different lineages of bats. Results of all tests 2 of the branch-site model A are shown in Table 2. No evidence of positive selection was detected on five focal branches leading to echolocating bats (branch A, B, C, D and E in Figure 1B) with the exception of the ancestral branch leading to yinpterochiropteran echolocating bats. Statistically supported evidence of positive selection (2Δ*ℓ* = 8.21, df = 1, *P = *0.004) was detected on the ancestral branch leading to Yinpterochiroptera echolocating bats, however, only one positively selected site was found [260Q, Bayes Empirical Bayes (BEB) values = 0.998] (Table 2). However, a strong signature of positive selection was detected by branch-site model A test on the ancestral branch of Old World fruit bats (2Δ*ℓ* = 4.39, df = 1, *P = *0.036) (Table 2). Twelve positively selected sites were detected on the ancestral branch of Old World fruit bats, of which two had BEB values >0.95 (677V and 1165I) (Table 2). As a comparison, we also performed the branch-site model A test on the ancestral branch of New World fruit bats, considering this taxon had evolved feeding habits similar to the Old World fruit bats. However, no evidence of positive selection was detected on that branch (Table 2). Alternative methods based on branch-site (REL) and branch (GA-branch) comparisons failed to detect positive selection in the Old World fruit bats and other branches tested above (data not shown).

**Table 2:** Results of branch-site model A tests for detection of positively selected sites in selected branches.

Branch-site model^a^	np^b^	Parameters	*ℓ*	*P*	Positively selected sites^c^
Null hypothesis for branch **A**	49	*P*_0_ = 0.966, *P*_1_ = 0.034, *P*_2a_ = 0.00, *P*_2b_ = 0.00Background: ω_0_ = 0.022, ω_1_ = 1.00, ω_2a_ = 0.022, ω_2b_ = 1.00Foreground: ω_0_ = 0.022, ω_1_ = 1.00, ω_2a_ = 1.00, ω_2b_ = 1.00	−15875.92	1	Not allowed
Alternative hypothesis for branch **A**	50	*P*_0_ = 0.966, *P*_1_ = 0.034, *P*_2a_ = 0.00, *P*_2b_ = 0.00Background: ω_0_ = 0.022, ω_1_ = 1.00, ω_2a_ = 0.022, ω_2b_ = 1.00Foreground: ω_0_ = 0.022, ω_1_ = 1.00, ω_2a_ = 1.00, ω_2b_ = 1.00	−15875.92		None
Null hypothesis for branch **B**	49	*P*_0_ = 0.966, *P*_1_ = 0.034, *P*_2a_ = 0.00, *P*_2b_ = 0.00Background: ω_0_ = 0.022, ω_1_ = 1.00, ω_2a_ = 0.022, ω_2b_ = 1.00Foreground: ω_0_ = 0.022, ω_1_ = 1.00, ω_2a_ = 1.00, ω_2b_ = 1.00	−15875.92	1	Not allowed
Alternative hypothesis for branch **B**	50	*P*_0_ = 0.966, *P*_1_ = 0.034, *P*_2a_ = 0.00, *P*_2b_ = 0.00Background: ω_0_ = 0.022, ω_1_ = 1.00, ω_2a_ = 0.022, ω_2b_ = 1.00Foreground: ω_0_ = 0.022, ω_1_ = 1.00, ω_2a_ = 1.00, ω_2b_ = 1.00	−15875.92		None
Null hypothesis for branch **C**	49	*P*_0_ = 0.966, *P*_1_ = 0.034, *P*_2a_ = 0.00, *P*_2b_ = 0.00Background: ω_0_ = 0.022, ω_1_ = 1.00, ω_2a_ = 0.022, ω_2b_ = 1.00Foreground: ω_0_ = 0.022, ω_1_ = 1.00, ω_2a_ = 1.00, ω_2b_ = 1.00	−15875.92	>0.05	Not allowed
Alternative hypothesis for branch **C**	50	*P*_0_ = 0.966, *P*_1_ = 0.034, *P*_2a_ = 0.00, *P*_2b_ = 0.00Background: ω_0_ = 0.022, ω_1_ = 1.00, ω_2a_ = 0.022, ω_2b_ = 1.00Foreground: ω_0_ = 0.022, ω_1_ = 1.00, ω_2a_ = 1.00, ω_2b_ = 1.00	−15875.92		None
Null hypothesis for branch **D**	49	*P*_0_ = 0.933, *P*_1_ = 0.033, *P*_2a_ = 0.033, *P*_2b_ = 0.001Background: ω_0_ = 0.022, ω_1_ = 1.00, ω_2a_ = 0.022, ω_2b_ = 1.00Foreground: ω_0_ = 0.022, ω_1_ = 1.00, ω_2a_ = 1.00, ω_2b_ = 1.00	−15874.88	0.004	Not allowed
Alternative hypothesis for branch **D**	50	*P*_0_ = 0.965, *P*_1_ = 0.034, *P*_2a_ = 0.001, *P*_2b_ = 0.00003Background: ω_0_ = 0.022, ω_1_ = 1.00, ω_2a_ = 0.022, ω_2b_ = 1.00Foreground: ω_0_ = 0.022, ω_1_ = 1.00, ω_2a_ = 238.51, ω_2b_ = 238.51	−15870.77		260Q (0.998)
Null hypothesis for branch **E**	49	*P*_0_ = 0.966, *P*_1_ = 0.034, *P*_2a_ = 0.00, *P*_2b_ = 0.00Background: ω_0_ = 0.022, ω_1_ = 1.00, ω_2a_ = 0.022, ω_2b_ = 1.00Foreground: ω_0_ = 0.022, ω_1_ = 1.00, ω_2a_ = 1.00, ω_2b_ = 1.00	−15875.92	>0.05	Not allowed
Alternative hypothesis for branch **E**	50	*P*_0_ = 0.966, *P*_1_ = 0.034, *P*_2a_ = 0.00, *P*_2b_ = 0.00Background: ω_0_ = 0.022, ω_1_ = 1.00, ω_2a_ = 0.022, ω_2b_ = 1.00Foreground: ω_0_ = 0.022, ω_1_ = 1.00, ω_2a_ = 1.00, ω_2b_ = 1.00	−15875.92		None
Null hypothesis for branch **F**	49	*P*_0_ = 0.907, *P*_1_ = 0.030, *P*_2a_ = 0.061, *P*_2b_ = 0.002Background: ω_0_ = 0.021, ω_1_ = 1.00, ω_2a_ = 0.021, ω_2b_ = 1.00Foreground: ω_0_ = 0.021, ω_1_ = 1.00, ω_2a_ = 1.00, ω_2b_ = 1.00	−15866.41	0.036	Not allowed
Alternative hypothesis for branch **F**	50	*P*_0_ = 0.954, *P*_1_ = 0.031, *P*_2a_ = 0.014, *P*_2b_ = 0.00046Background: ω_0_ = 0.021, ω_1_ = 1.00, ω_2a_ = 0.021, ω_2b_ = 1.00Foreground: ω_0_ = 0.021, ω_1_ = 1.00, ω_2a_ = 6.679, ω_2b_ = 6.679	−15864.21		147V, 300H, 521S, 534S, 560V, 677V (0.981), 678G, 791Y, 903V, 913G, 1165I (0.972), 1169K
Null hypothesis for branch **G**	49	*P*_0_ = 0.966, *P*_1_ = 0.034, *P*_2a_ = 0.00, *P*_2b_ = 0.00Background: ω_0_ = 0.022, ω_1_ = 1.00, ω_2a_ = 0.022, ω_2b_ = 1.00Foreground: ω_0_ = 0.022, ω_1_ = 1.00, ω_2a_ = 1.00, ω_2b_ = 1.00	−15875.92	1	Not allowed
Alternative hypothesis for branch **G**	50	*P*_0_ = 0.966, *P*_1_ = 0.034, *P*_2a_ = 0.00, *P*_2b_ = 0.00Background: ω_0_ = 0.022, ω_1_ = 1.00, ω_2a_ = 0.022, ω_2b_ = 1.00Foreground: ω_0_ = 0.022, ω_1_ = 1.00, ω_2a_ = 1.00, ω_2b_ = 1.00	−15875.92		None

a See Figure 1B for branch labels.

b np, number of parameters.

c Positively selected sites detected by branch-site model A test are referred to *Megaderma lyra* in branch D and to *Rousettus leschenaultii* in branch F, respectively. Sites with Posterior probability values >0.95 are highlighted with underline. Site positions were referred to the *Myo6* amino acid sequence alignment of all species (Figure S2 and Figure S3).

**Figure 2:**
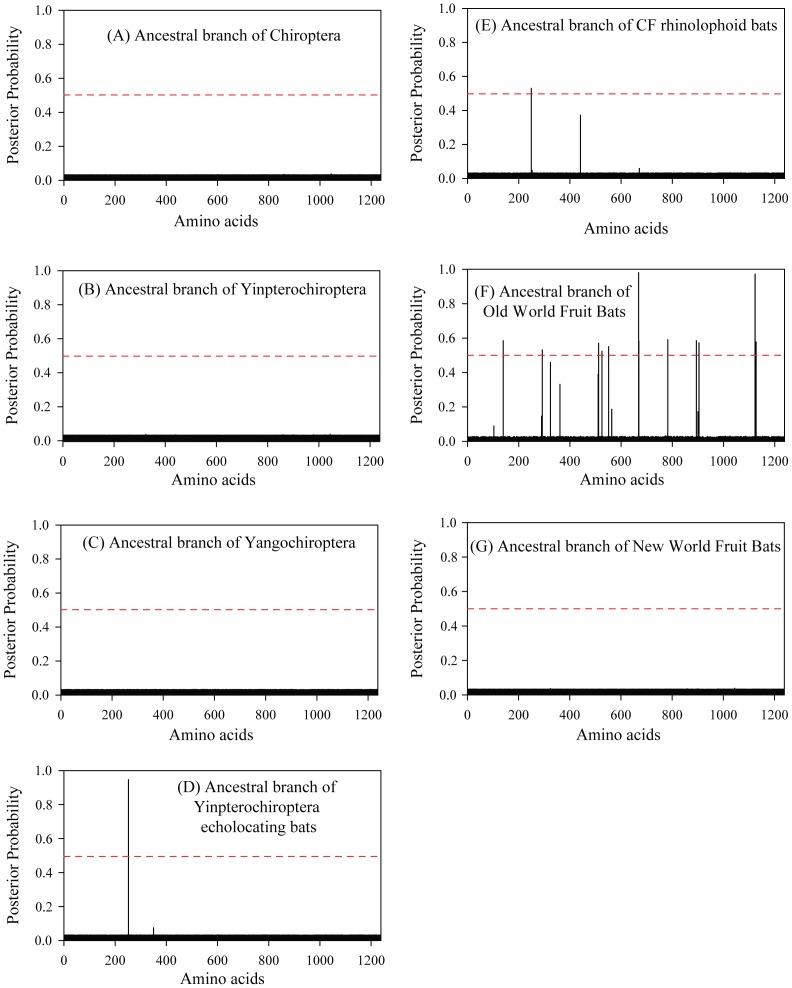
Site-wise posterior probabilities of positive selection for sites along the *Myo6* sequence of selected branches. The posterior probability value of 0.5 is indicated by the red dashed line in each plot.

We also calculated the posterior probabilities of positively selected sites for each amino acid of *Myo6* on the above seven branches which tested both by two-ratio model tests and branch-site model A tests. In accordance with the results of branch-site model tests, significant evidence of positive selection was found on ancestral branch leading to Old World fruit bats (Figure 2).

**Figure 3:**
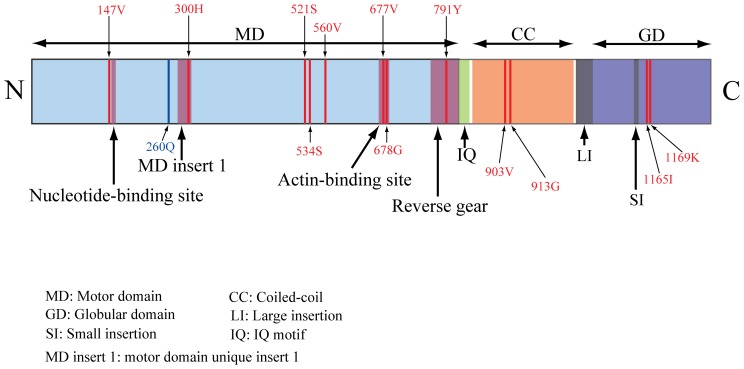
Distribution of positively selected sites detected on the ancestral branches of Yinpterochiroptera echolocating bats and the Old World fruit bats in the secondary structure of myosin VI protein. Protein structure was constructed based on previous studies (see Results). The positively selected site found on the ancestral branches of Yinpterochiroptera echolocating bats is highlighted with blue line. Twelve positively selected sites detected in the Old World fruit bats are highlighted with red lines.

In order to examine the distribution pattern and potential influences of the positively selected sites detected in the ancestral branches leading to yinpterochiropteran echolocating bats (one amino acid site) and Old World fruit bats (12 amino acid sites), respectively, we mapped these amino acid sites onto the myosin VI protein secondary structure constructed in previous studies [18], [65], [66], [67], [68]. Of the 13 positively selected sites, nine were distributed on the motor domain (147V, 260Q, 300H, 521S, 534S, 560V, 677V, 678G and 791Y), two on the coiled-coil region (903V and 913G) and two on the globular domain after large and small insertions (1165I and 1169K) (Figure 3).

**Figure 4:**
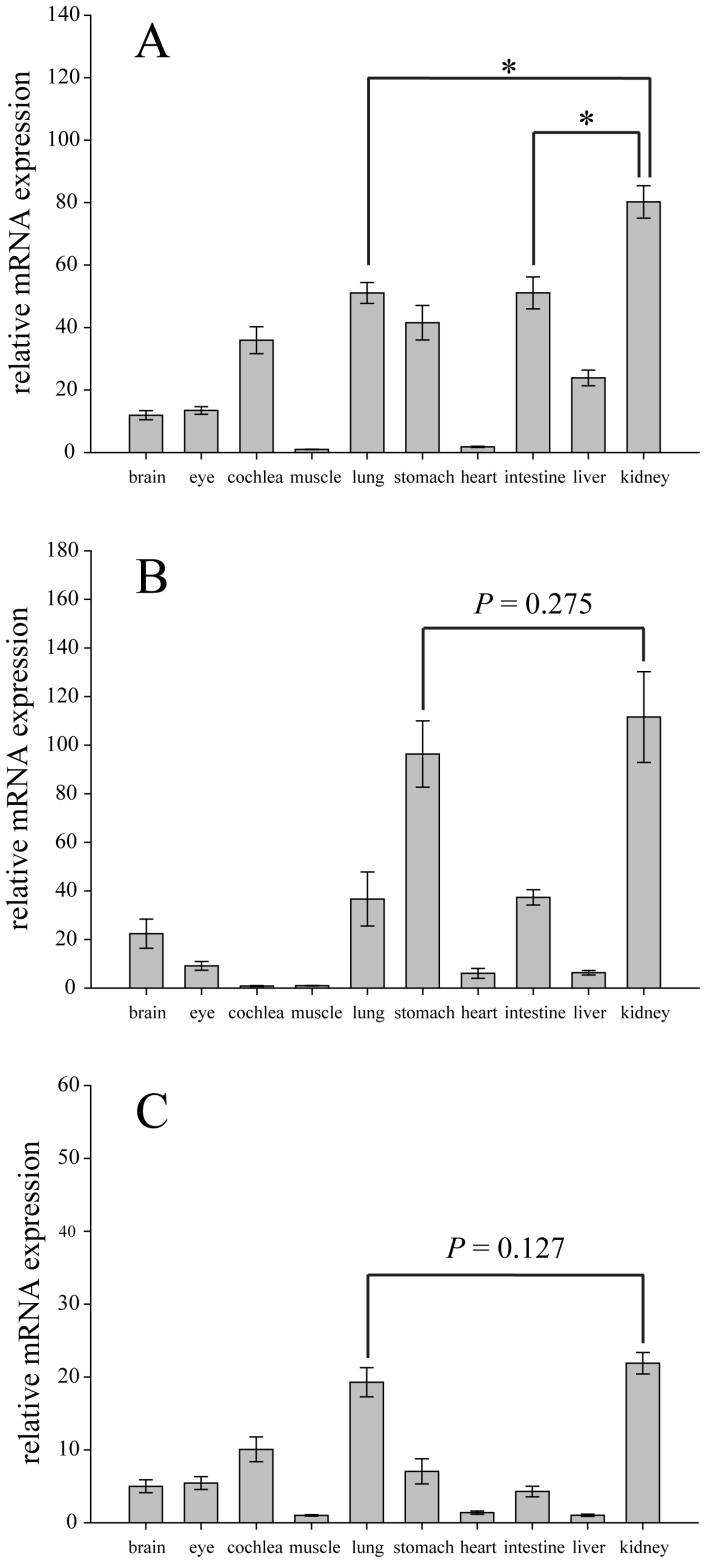
Expression levels of *Myo6* mRNA in ten tissues among three representative bat species. (**A**) *Cynopterus sphinx*, (**B**) *Rhinolophus ferrumequinum* and (**C**) *Myotis ricketti*. The amount of *Myo6* in each tissue were normalized to the *Gapdh* gene and arbitrarily calibrated to muscle for each species using the 2^−ΔΔCt^ method. For each species, three individuals were used for replication. Error bars show the standard deviation (SD). The asterisk (*) indicates *P*-value <0.05 (two-tailed nonparametric Mann-Whitney U test).

We also performed Real-time PCR assays to determine the expression of *Myo6* mRNA in ten tissues of three representative bat species: *C*. *sphinx*, *R*. *ferrumequinum* and *M*. *ricketti*. *Myo6* was ubiquitously expressed in all these ten tissues of three bat species. For *C*. *sphinx*, expression levels of the *Myo6* gene among ten tissues were significantly different (*P*<0.001, df = 9, Kruskal-wallis test), and the highest expression of the *Myo6* gene occurred in the kidney (Figure 4A). For *R*. *ferrumequinum* and *M*. *ricketti*, the *Myo6* expression levels within ten tissues were also significantly different (*P*<0.001 and *P*<0.01, respectively, df = 9, Kruskal-wallis test), and the highest expression of the *Myo6* gene were also found in the kidney (Figure 4B and 4C). However, for *R*. *ferrumequinum*, the expression of the *Myo6* in the kidney was not significantly higher than that of the stomach (*P* = 0.275, Mann-Whitney U test) (Figure 4B). And for *M*. *ricketti*, the expression of the gene in the kidney was not significantly higher than that of the lung (*P* = 0.127, Mann-Whitney U test) (Figure 4C). These results indicating that the *Myo6* gene might play an important role in kidney function, especially in the frugivorous Old World fruit bat, *C*. *sphinx*.

## Discussion

Results from two-ratio model tests showed that no selection pressure change was found in major focal branches leading to echolocating bats and also the New World fruit bats. However, the results of two-ratio model tests revealed a significantly greater value of ω (d_N_/d_S_) on the ancestral branch of Old World fruit bats compared with the rest of the tree, indicating a selection pressure change acting on *Myo6* in this lineage. The subsequent branch-site model A test revealed that *Myo6* has undergone adaptive evolution in the ancestral branch leading to Old World fruit bats.

Our results of real-time PCR showed the highest expression of *Myo6* gene in the kidney in *C*. *sphinx* (Figure 4), indicating that proteins produced by this gene may be involved in kidney function in frugivorous Old World fruit bats. However, we could not show the expression levels of *Myo6* isoforms separately in each tissue, since the primers we used were designed based on the sequences of the conserved tail domain after both large and small insertions. However the primer design is unlikely to influence *Myo6* expression patterns shown with our real-time PCR results, considering the *Myo6* isoforms with 32aa large insertion and 9aa small insertion were specifically expressed in polarized and unpolarized cells, respectively [25]. Our results show discrepancies with a previous study, in which the highest levels of *Myo6* expression was found in the brain, pancreas, prostate, testis and small intestine but not in the kidney of human tissues studied by northern blot methods [5]. The discrepancy might be caused by methodological differences, but is more likely to reflect differences in expression patterns in bats compared with humans.

That the *Myo6* gene functions in hearing is supported by overwhelming evidence, as mutations cause non-syndromic deafness in humans [5], [6], [7], [69]. The evolution of echolocation and its associated high-frequency hearing makes laryngeal echolocating bats a fascinating mammal group for molecular evolutionary studies of hearing genes. Many genes associated with hearing have recently been proved to have undergone positive selection in echolocating bats and also in echolocating cetaceans [10], [11], [12], [13], [14], [15], [16], [70]. However, our results from the branch-site model A tests showed no significant evidence of positive selection in the major focal branches leading to echolocating bats, although one statistically supported positively selected site (260Q) was found in the ancestral branch of Yinpterochiroptera echolocating bats (Table 2). These results indicate that although the *Myo6* gene is fundamental for the development and maintenance of stereocilia [2], [4], [5], this gene was not a potential target of positive selection and thus may not contribute to the evolution of high-frequency hearing in echolocating bats.

On the contrary, our results of branch-site model A tests revealed strong evidence for positive selection acting on the ancestral branch of Old World fruit bats, a lineage that does not possess laryngeal echolocation [8]. These results suggest that the *Myo6* gene may be involved in other important physiological functions rather than hearing in Old World fruit bats. Some studies have reported that the *Myo6* gene is also abundantly expressed in the retina and is required for the normal functioning of photoreceptor cells [17], [18]. In accordance with these observations, our real-time PCR assays also detected the expression of the *Myo6* gene in the eyes of *C*. *sphinx*, *R*. *ferrumequinum* and *M*. *ricketti*, although the expression levels were all relatively low (Figure 4). Since they lack the ability of laryngeal echolocation, Old World fruit bats are more dependent on vision for perceiving their environment than are their echolocating relatives [19], [20], [21]. Thus we could not role out the possibility that the positive selection of the *Myo6* gene may have been driven by the requirement for more effective vision in Old World fruit bats. In photoreceptors, the *Myo6* gene is highly expressed in the margins of the inner segments and the outer surface of the ellipsoid mitochondrial mass [17], [18]. *Myo6* gene might be involved in the localization of mitochondria which may be important for dark current generation by the sufficient supply of ATP to Na^+^, K^+^-ATPases on the inner segment plasma membrane [17], [18]. In retinal pigment epithelial (RPE) cells, *Myo6* might play an important role in transportation of lysosomes which are responsible for the normal daily digestion of photoreceptor disc membrane [17]. Considering those potential important roles of the *Myo6* gene in retinal, we could not role out the possibility that the positive selection of this gene in Old World fruit bats might relate to the evolution of their effective visual system to enhance their visual sensitivity at dim-light environment. Because the evidence for the exact role that *Myo6* plays in vision is still unclear, more studies on its function are needed to determine the possible role of this gene in vision in Old World fruit bats.

A large number of studies have recently identified an important role for myosin VI in clathrin-mediated endocytosis in polarized epithelial cells such as kidney proximal tubule cells and intestinal enterocytes (see Introduction). Thus another possible explanation for positive selection acting on *Myo6* in Old World fruit bats is the adaptation of receptor-mediated endocytosis in kidney proximal tubule cells. *Myo6* is highly expressed in the kidney where it located in the brush border of renal proximal tubule cells [24], which is also supported by our real-time PCR results (Figure 4). Moreover, the study of myosin VI functional null *Snell’s waltzer* (*sv*/*sv*) mice revealed that myosin VI plays an important role in kidney proximal tubule protein reabsorption, as *sv*/*sv* mice showed an albuminuria phenotype [27]. The significantly high expression of the *Myo6* gene in the kidney of *C*. *sphinx* (Figure 4A) strongly indicated the important role of this gene in kidney function in Old World fruit bats. Although some studies observed that Old World fruit bats accidentally or even deliberately consume insects, a predominantly frugivorous food habit has recently been confirmed by studies with stable-isotope analyses (see Introduction). Thus, the low protein content in the special diets of Old World fruit bats [37], [42], [71] highlights the significance of protein preservation by this mechanism in Old World fruit bats compared with insectivorous, omnivorous and carnivorous bats. Although renal proximal tubule reabsorption is highly efficient, small quantities of protein would still naturally be lost in urine in healthy humans and other animals [72], [73], [74]. Thus, it is plausible that Old World fruit bats have evolved a more efficient receptor-mediated endocytosis for protein preservation to adapt to their low protein diets.

Alternatively, the positive selection of *Myo6* in Old World fruit bats might relate to the preservation of essential nutrients such as vitamin D [25-(OH) vitamin D_3_] and vitamin B_12_ rather than proteins. 25-(OH) vitamin D_3_ is filtered by glomeruli as a complex with vitamin D-binding protein (DBP) and then reabsorbed into proximal tubule cells by receptor-mediated endocytosis [26], [28], [30]. Then 25-(OH) vitamin D_3_ is transported to mitochondria and hydroxylated into 1,25-(OH)_2_ vitamin D_3_, which is the active form of vitamin D for the regulation of metabolism [28], [30], [75]. Thus, receptor-mediated endocytosis is of great importance for the preservation and activation of vitamin D, since defects in this process would cause vitamin D deficiency and disease [28], [30]. Similarly, vitamin B_12_ is filtered and then reabsorbed as a complex with transcobalamin (TC) [31]. The reabsorption and accumulation of vitamin B_12_ by receptor-mediated endocytosis is of great importance for vitamin B_12_ homeostasis [26], [31], [76]. Since fruit is known to be devoid of both these fat-soluble vitamin components [39], Old World fruit bats (especially frugivorous bats) are thought to be naturally in a state of vitamin D and vitamin B_12_ deficiency [77], [78], [79]. Thus, it is reasonable to assume that the adaptive change of *Myo6* in Old World fruit bats might relate to the evolution of their efficient receptor-mediated endocytosis for the preservation and homeostasis of essential fat-soluble vitamins or other similar components which are deficient in plant food resources.

The 12 positively selected sites detected by branch-site model A tests in the Old World fruit bats (Table 2) are mainly distributed in the motor domain region (eight sites), the coiled-coil domain (two sites) and the globular tail domain (two sites) (Figure 3). The motor domain is known to be important for the minus-end directed movement [1], [80], and mutations in this domain could cause motor activity impairment and subsequent transportation blocking [81], [82], [83]. Notably, for these eight sites distributed in the motor domain region, we found that the residue 147V was located close to the nucleotide-binding site and 677V and 678G were within the predicted actin-binding site (Figure 3). Residue 300H was distributed in the putative motor domain unique insert 1 region, which is thought to be involved in the modulation of nucleotide binding and release [67]. Residue 791Y was distributed in the reverse gear region which is involved in the lever-arm redirection and thus responsible for the reverse movement of myosin VI [66], [67]. The coiled-coli region is thought to mediate the dimerization of myosin VI [84], and the globular tail domain is necessary for cargo-targeting [85]. Thus, the positively selected sites might influence the activities of these regions and therefore the efficiency of the myosin VI protein. Among these 12 positively selected sites, nine amino acid changes at positions 147, 534, 560, 677, 678, 791, 903, 913 and 1169 were found to be specific to the Old World fruit bats compared with other bats and mammal groups (Figure S3). The amino acid change from hydrophilic Threonine (T) to hydrophobic Valine (V) at position 677 (Figure S3) might enhance the capability of binding/recognition of hydrophobic ligands [86]. The amino acid change from Cysteine (C) to Tyrosine (Y) at position 791 (Figure S3) involved the introduction of an extra aromatic side chain, and might enhance the ability of stacking interactions with other aromatic side chain [86]. The amino acid change from Glutamine (Q) to Lysine (K) at position 1169 (Figure S3) introduced a positively-charged amino group, and might be involved in the binding of negatively-charged ligands [86]. In the absence of direct biochemical evidence, we are unable to assign more significance to these amino acid changes in the Old World fruit bats. More studies are needed to determine what functional changes in the myosin VI protein may result from mutations at these sites.

In addition, the New World fruit bats which belong to the family Phyllostomidae, especially the members of the subfamily Stenodermatinae, have also evolved a predominantly frugivorous feeding habit similar to that of Old World fruit bats [32]. However, no positively selected site was detected on the ancestral branch of this lineage (Table 2), and also in the species of *A. lituratus* which belongs to the subfamily Stenodermatinae (data not shown). These results might reflect the fact that the Old World fruit bats evolved such special diets slightly earlier than did the New World fruit bats (nearly 28mya versus almost 20mya) [56]. Moreover, another plausible explanation for these results might be that fruit available to the Old World fruit bats and the New World fruit bats was different during the time of their radiations. Neotropical regions possess a greater diversity of food plants and more stable plant food resources than do Palaeotropical regions [87]. Thus it is possible for species of New World fruit bats to selectively ingest mixed fruit species to obtain sufficient proteins [88] without the evolution of an adaptive mechanism for the preservation of protein and essential nutrients as found in Old World fruit bats. It is interesting to note that, other molecular evolutionary studies focusing on genes in bats (and other mammals) in relation to their diets also show a similar discrepancy between the Old World and the New World fruit bats [89], [90]. For instance, the glucose transporter 4 (GLUT4, encoded by *Slc2a4*) which plays a crucial role in glucose homeostasis has undergone adaptive changes only in the Old World fruit bats in relation to their high sugar diet but not in the New World fruit bats [89].

In conclusion, our results show that the *Myo6* gene, which was widely considered as a hearing gene, has undergone adaptive evolution in the Old World fruit bats without laryngeal echolocation and associated high-frequency hearing. Positive selection on *Myo6* in Old World fruit bats may be related to their specialized diets. In combination with the high levels of expression of *Myo6* in kidney tissue, our results provide evidence that *Myo6* has undergone adaptive evolution in Old World fruit bats in relation to receptor-mediated endocytosis for the preservation of protein and essential nutrients. As receptor mediated-endocytosis is a multistep process and involves many molecules other than myosin VI [2], [3], [66], more studies are needed to delineate what genes in addition to *Myo6* may also contribute to the specialized dietary adaptations of Old World fruit bats.

## Supporting Information

Figure S1 **Myosin VI protein structure showing the differences of** ***Myo6*** **isoforms from brain and kidney.** (a) A cartoon illustrating the four overlapped fragments for PCR. The *Myo6* coding sequence was divided into four overlapped fragments, for each fragment a pair of primers were designed for amplification. (b) Schematic of myosin VI structure with the large and small insertions in tail domain are shown. (c) Comparison of amino acid sequences of *Myo6* isoforms from brain and kidney. The sequences framed are the large insertion (32aa) in the kidney isoform and the small insertion (9aa) in the brain isoform.
(TIF)

Figure S2 **Alignment of the amino acid sequences of the** ***Myo6*** **gene from 24 mammals (only the variable sites are shown).**
(TIF)

Figure S3 **Alignment of the full amino acid sequences of the** ***Myo6*** **gene from 24 mammals.** Twelve positively selected sites detected in the Old World fruit bats are highlighted by red squares and indicated with asterisks on above of alignment columns.
(PDF)

Table S1 List of species analyzed in this study.
(DOC)

Table S2 Information of primers used for *Myo6* coding sequences PCR and real-time PCR.
(DOC)

## References

[1] Wells AL, Lin AW, Chen LQ, Safer D, Cain SM, et al (1999) Myosin VI is an actin-based motor that moves backwards. Nature 401: 505–508. 10519557 10.1038/46835

[2] Buss F, Spudich G, Kendrick-Jones J (2004) Myosin VI: cellular functions and motor properties. Annu Rev Cell Dev Biol 20: 649–676. 15473855 10.1146/annurev.cellbio.20.012103.094243

[3] Hasson T (2003) Myosin VI: two distinct roles in endocytosis. J Cell Sci 116: 3453–3461. 12893809 10.1242/jcs.00669

[4] Self T, Sobe T, Copeland NG, Jenkins NA, Avraham KB, et al (1999) Role of myosin VI in the differentiation of cochlear hair cells. Dev Biol 214: 331–341. 10525338 10.1006/dbio.1999.9424

[5] Avraham KB, Hasson T, Sobe T, Balsara B, Testa JR, et al (1997) Characterization of unconventional MYO6, the human homologue of the gene responsible for deafness in Snell’s waltzer mice. Hum Mol Genet 6: 1225–1231. 9259267 10.1093/hmg/6.8.1225

[6] Ahmed ZM, Morell RJ, Riazuddin S, Gropman A, Shaukat S, et al (2003) Mutations of MYO6 are associated with recessive deafness, DFNB37. Am J Hum Genet 72: 1315–1322. 12687499 10.1086/375122 PMC1180285

[7] Melchionda S, Ahituv N, Bisceglia L, Sobe T, Glaser F, et al (2001) MYO6, the human homologue of the gene responsible for deafness in Snell’s waltzer mice, is mutated in autosomal dominant nonsyndromic hearing loss. Am J Hum Genet 69: 635–640. 11468689 10.1086/323156 PMC1235492

[8] Jones G, Teeling EC (2006) The evolution of echolocation in bats. Trends Ecol Evol 21: 149–156. 16701491 10.1016/j.tree.2006.01.001

[9] Fenton MB, Portfors CV, Rautenbach IL, Waterman JM (1998) Compromises: sound frequencies used in echolocation by aerial-feeding bats. Can J Zool 76: 1174–1182.

[10] Rossiter SJ, Zhang S, Liu Y (2011) Prestin and high frequency hearing in mammals. Commun Integr Biol 4: 236–239. 21655450 10.4161/cib.4.2.14647 PMC3104589

[11] Liu Z, Li S, Wang W, Xu D, Murphy RW, et al (2011) Parallel evolution of *KCNQ4* in echolocating bats. PLoS One 6: e26618. 22046315 10.1371/journal.pone.0026618 PMC3200345

[12] Liu Y, Han N, Franchini LF, Xu H, Pisciottano F, et al (2011) The voltage-gated potassium channel subfamily KQT member 4 (*KCNQ4*) displays parallel evolution in echolocating bats. Mol Biol Evol 29: 1441–1450. 22319145 10.1093/molbev/msr310 PMC3339320

[13] Liu Y, Rossiter SJ, Han X, Cotton JA, Zhang S (2010) Cetaceans on a molecular fast track to ultrasonic hearing. Curr Biol 20: 1834–1839. 20933423 10.1016/j.cub.2010.09.008

[14] Liu Y, Cotton JA, Shen B, Han X, Rossiter SJ, et al (2010) Convergent sequence evolution between echolocating bats and dolphins. Curr Biol 20: R53–54. 20129036 10.1016/j.cub.2009.11.058

[15] Li Y, Liu Z, Shi P, Zhang J (2010) The hearing gene *Prestin* unites echolocating bats and whales. Curr Biol 20: R55–56. 20129037 10.1016/j.cub.2009.11.042 PMC11646320

[16] Li G, Wang J, Rossiter SJ, Jones G, Cotton JA, et al (2008) The hearing gene *Prestin* reunites echolocating bats. Proc Natl Acad Sci U S A 105: 13959–13964. 18776049 10.1073/pnas.0802097105 PMC2544561

[17] Kitamoto J, Libby RT, Gibbs D, Steel KP, Williams DS (2005) Myosin VI is required for normal retinal function. Exp Eye Res 81: 116–120. 15978262 10.1016/j.exer.2005.02.014

[18] Breckler J, Au K, Cheng J, Hasson T, Burnside B (2000) Novel myosin VI isoform is abundantly expressed in retina. Exp Eye Res 70: 121–134. 10644428 10.1006/exer.1999.0758

[19] Heffner RS, Koay G, Heffner HE (2007) Sound-localization acuity and its relation to vision in large and small fruit-eating bats: I. Echolocating species, *Phyllostomus hastatus* and *Carollia perspicillata* . Hear Res 234: 1–9. 17630232 10.1016/j.heares.2007.06.001 PMC2141540

[20] Heffner RS, Koay G, Heffner HE (2001) Sound localization in a new-world frugivorous bat, *Artibeus jamaicensis*: acuity, use of binaural cues, and relationship to vision. J Acoust Soc Am 109: 412–421. 11206172 10.1121/1.1329620

[21] Fuzessery ZM, Buttenhoff P, Andrews B, Kennedy JM (1993) Passive sound localization of prey by the pallid bat (*Antrozous p. pallidus*). J Comp Physiol A 171: 767–777. 8441123 10.1007/BF00213073

[22] Raghuram H, Thangadurai C, Gopukumar N, Nathar K, Sripathi K (2009) The role of olfaction and vision in the foraging behaviour of an echolocating megachiropteran fruit bat, *Rousettus leschenaulti* (Pteropodidae). Mamm Biol 74: 9–14.

[23] Ameen N, Apodaca G (2007) Defective CFTR apical endocytosis and enterocyte brush border in myosin VI-deficient mice. Traffic 8: 998–1006. 17555536 10.1111/j.1600-0854.2007.00587.x

[24] Biemesderfer D, Mentone SA, Mooseker M, Hasson T (2002) Expression of myosin VI within the early endocytic pathway in adult and developing proximal tubules. Am J Physiol Renal Physiol 282: F785–794. 11934687 10.1152/ajprenal.00287.2001

[25] Buss F, Arden SD, Lindsay M, Luzio JP, Kendrick-Jones J (2001) Myosin VI isoform localized to clathrin-coated vesicles with a role in clathrin-mediated endocytosis. EMBO J 20: 3676–3684. 11447109 10.1093/emboj/20.14.3676 PMC125554

[26] Christensen EI, Verroust PJ, Nielsen R (2009) Receptor-mediated endocytosis in renal proximal tubule. Pflugers Arch 458: 1039–1048. 19499243 10.1007/s00424-009-0685-8

[27] Gotoh N, Yan Q, Du Z, Biemesderfer D, Kashgarian M, et al (2010) Altered renal proximal tubular endocytosis and histology in mice lacking myosin-VI. Cytoskeleton (Hoboken) 67: 178–192. 20175219 10.1002/cm.20435 PMC3468331

[28] Nykjaer A, Fyfe JC, Kozyraki R, Leheste JR, Jacobsen C, et al (2001) Cubilin dysfunction causes abnormal metabolism of the steroid hormone 25(OH) vitamin D_3_ . Proc Natl Acad Sci U S A 98: 13895–13900. 11717447 10.1073/pnas.241516998 PMC61138

[29] Kozyraki R, Fyfe J, Verroust PJ, Jacobsen C, Dautry-Varsat A, et al (2001) Megalin-dependent cubilin-mediated endocytosis is a major pathway for the apical uptake of transferrin in polarized epithelia. Proc Natl Acad Sci U S A 98: 12491–12496. 11606717 10.1073/pnas.211291398 PMC60081

[30] Nykjaer A, Dragun D, Walther D, Vorum H, Jacobsen C, et al (1999) An endocytic pathway essential for renal uptake and activation of the steroid 25-(OH) vitamin D_3_ . Cell 96: 507–515. 10052453 10.1016/s0092-8674(00)80655-8

[31] Moestrup SK, Birn H, Fischer PB, Petersen CM, Verroust PJ, et al (1996) Megalin-mediated endocytosis of transcobalamin-vitamin-B_12_ complexes suggests a role of the receptor in vitamin-B_12_ homeostasis. Proc Natl Acad Sci U S A 93: 8612–8617. 8710919 10.1073/pnas.93.16.8612 PMC38721

[32] Voigt CC, Zubaid A, Kunz TH, Kingston T (2011) Sources of assimilated proteins in Old and New World phytophagous bats. Biotropica 43: 108–113.

[33] Herrera GL, Gutierrez E, Hobson KA, Altube B, Díaz WG, et al (2002) Sources of assimilated protein in five species of New World frugivorous bats. Oecologia 133: 280–287. 28466224 10.1007/s00442-002-1036-z

[34] Herrera MLG, Hobson KA, Mirón ML, Ramírez PN, Méndez CG, et al (2001) Sources of protein in two species of phytophagous bats in a seasonal dry forest: evidence from stable-isotope analysis. J Mamm 82: 352–361.

[35] Barclay RMR, Barclay LE, Jacobs DS (2006) Deliberate insectivory by the fruit bat *Rousettus aegyptiacus* . Acta Chiropt 8: 549–553.

[36] Parry-Jones K, Augee ML (1992) Insects in flying fox diets. Bat Res News 33: 9–11.

[37] Neuweiler G (2000) The biology of bats. New York: Oxford University Press.

[38] Gottsberger G, Schrauwen J, Linskens HF (1984) Amino acids and sugars in nectar, and their putative evolutionary significance. Plant Syst Evol 145: 55–77.

[39] Holland B, Welch AA, Unwin ID, Buss DH, Paul AA, et al. (1992) McCance and Widdowson’s The composition of foods. Letchworth, UK: Royal Society of Chemistry.

[40] van der Westhuyzen J, Cantrill RC, Fernandes-Costa F, Metz J (1982) Lipid composition of the brain in the vitamin B_12_-deficient fruit bat (*Rousettus aegyptiacus*) with neurological impairment. J Neurochem 37: 543–549. 10.1111/j.1471-4159.1982.tb12521.x 7276938

[41] Buckland-Wright JC, Pye JD (1973) Dietary deficiency in fruit bats. International Zoo Yearbook 13: 271–277.

[42] Courts SE (1998) Dietary strategies of Old World fruit bats (Megachiroptera, Pteropodidae): how do they obtain sufficient protein? Mamm Rev 28: 185–194.

[43] Tedman RA, Hall LS (1985) The morphology of the gastrointestinal tract and food transit time in the fruit bats *Pteropus alecto* and *P. poliocephalus* (Megachiroptera). Aust J Zool 33: 625–640.

[44] Thomas DW (1984) Fruit intake and energy budgets of frugivorous bats. Physiol Zool 57: 457–467.

[45] Shen B, Avila-Flores R, Liu Y, Rossiter SJ, Zhang S (2011) *Prestin* shows divergent evolution between constant frequency echolocating bats. J Mol Evol 73: 109–115. 21947331 10.1007/s00239-011-9460-5

[46] Thompson JD, Gibson TJ, Plewniak F, Jeanmougin F, Higgins DG (1997) The CLUSTAL_X windows interface: flexible strategies for multiple sequence alignment aided by quality analysis tools. Nucleic Acids Res 25: 4876–4882. 9396791 10.1093/nar/25.24.4876 PMC147148

[47] Tamura K, Dudley J, Nei M, Kumar S (2007) MEGA4: Molecular Evolutionary Genetics Analysis (MEGA) software version 4.0. Mol Biol Evol 24: 1596–1599. 17488738 10.1093/molbev/msm092

[48] Ronquist F, Huelsenbeck JP (2003) MrBayes 3: Bayesian phylogenetic inference under mixed models. Bioinformatics 19: 1572–1574. 12912839 10.1093/bioinformatics/btg180

[49] Posada D (2008) jModelTest: phylogenetic model averaging. Mol Biol Evol 25: 1253–1256. 18397919 10.1093/molbev/msn083

[50] Posada D, Crandall KA (2002) The effect of recombination on the accuracy of phylogeny estimation. J Mol Evol 54: 396–402. 11847565 10.1007/s00239-001-0034-9

[51] Shriner D, Nickle DC, Jensen MA, Mullins JI (2003) Potential impact of recombination on sitewise approaches for detecting positive natural selection. Genet Res 81: 115–121. 12872913 10.1017/s0016672303006128

[52] Kosakovsky Pond SL, Posada D, Gravenor MB, Woelk CH, Frost SD (2006) GARD: a genetic algorithm for recombination detection. Bioinformatics 22: 3096–3098. 17110367 10.1093/bioinformatics/btl474

[53] Pond SL, Frost SD, Muse SV (2005) HyPhy: hypothesis testing using phylogenies. Bioinformatics 21: 676–679. 15509596 10.1093/bioinformatics/bti079

[54] Zhou X, Xu S, Xu J, Chen B, Zhou K, et al (2012) Phylogenomic analysis resolves the interordinal relationships and rapid diversification of the Laurasiatherian mammals. Syst Biol 61: 150–164. 21900649 10.1093/sysbio/syr089 PMC3243735

[55] Datzmann T, von Helversen O, Mayer F (2010) Evolution of nectarivory in phyllostomid bats (Phyllostomidae Gray, 1825, Chiroptera: Mammalia). BMC Evol Biol 10: 165. 20525339 10.1186/1471-2148-10-165 PMC2901259

[56] Teeling EC, Springer MS, Madsen O, Bates P, O’Brien SJ, et al (2005) A molecular phylogeny for bats illuminates biogeography and the fossil record. Science 307: 580–584. 15681385 10.1126/science.1105113

[57] Yang Z (2007) PAML 4: phylogenetic analysis by maximum likelihood. Mol Biol Evol 24: 1586. 17483113 10.1093/molbev/msm088

[58] Jones G (1999) Scaling of echolocation call parameters in bats. J Exp Biol 202: 3359–3367. 10562518 10.1242/jeb.202.23.3359

[59] Zhang J, Nielsen R, Yang Z (2005) Evaluation of an improved branch-site likelihood method for detecting positive selection at the molecular level. Mol Biol Evol 22: 2472–2479. 16107592 10.1093/molbev/msi237

[60] Kosakovsky Pond SL, Murrell B, Fourment M, Frost SD, Delport W, et al (2011) A random effects branch-site model for detecting episodic diversifying selection. Mol Biol Evol 28: 3033–3043. 21670087 10.1093/molbev/msr125 PMC3247808

[61] Pond SL, Frost SD (2005) A genetic algorithm approach to detecting lineage-specific variation in selection pressure. Mol Biol Evol 22: 478–485. 15509724 10.1093/molbev/msi031

[62] Barber RD, Harmer DW, Coleman RA, Clark BJ (2005) GAPDH as a housekeeping gene: analysis of GAPDH mRNA expression in a panel of 72 human tissues. Physiol Genomics 21: 389–395. 15769908 10.1152/physiolgenomics.00025.2005

[63] Vandesompele J, De Preter K, Pattyn F, Poppe B, Van Roy N, et al (2002) Accurate normalization of real-time quantitative RT-PCR data by geometric averaging of multiple internal control genes. Genome Biol 3: RESEARCH0034. 12184808 10.1186/gb-2002-3-7-research0034 PMC126239

[64] Livak KJ, Schmittgen TD (2001) Analysis of relative gene expression data using real-time quantitative PCR and the 2^−ΔΔCt^ method. Methods 25: 402–408. 11846609 10.1006/meth.2001.1262

[65] Heidrych P, Zimmermann U, Kuhn S, Franz C, Engel J, et al (2009) Otoferlin interacts with myosin VI: implications for maintenance of the basolateral synaptic structure of the inner hair cell. Hum Mol Genet 18: 2779–2790. 19417007 10.1093/hmg/ddp213

[66] Buss F, Kendrick-Jones J (2008) How are the cellular functions of myosin VI regulated within the cell? Biochem Biophys Res Commun 369: 165–175. 18068125 10.1016/j.bbrc.2007.11.150 PMC2635068

[67] Ménétrey J, Bahloul A, Wells AL, Yengo CM, Morris CA, et al (2005) The structure of the myosin VI motor reveals the mechanism of directionality reversal. Nature 435: 779–785. 15944696 10.1038/nature03592 PMC2762700

[68] Dance AL, Miller M, Seragaki S, Aryal P, White B, et al (2004) Regulation of myosin-VI targeting to endocytic compartments. Traffic 5: 798–813. 15355515 10.1111/j.1600-0854.2004.00224.x

[69] Hilgert N, Topsakal V, van Dinther J, Offeciers E, Van de Heyning P, et al (2008) A splice-site mutation and overexpression of MYO6 cause a similar phenotype in two families with autosomal dominant hearing loss. Eur J Hum Genet 16: 593–602. 18212818 10.1038/sj.ejhg.5202000

[70] Davies KT, Cotton JA, Kirwan JD, Teeling EC, Rossiter SJ (2012) Parallel signatures of sequence evolution among hearing genes in echolocating mammals: an emerging model of genetic convergence. Heredity 108: 480–489. 22167055 10.1038/hdy.2011.119 PMC3330687

[71] Wendeln MC, Runkle JR, Kalko EKV (2000) Nutritional values of 14 fig species and bat feeding preferences in Panama. Biotropica 32: 489–501.

[72] Uberti B, Eberle DB, Pressler BM, Moore GE, Sojka JE (2009) Determination of and correlation between urine protein excretion and urine protein-to-creatinine ratio values during a 24-hour period in healthy horses and ponies. Am J Vet Res 70: 1551–1556. 19951128 10.2460/ajvr.70.12.1551

[73] Novick D, Engelmann H, Wallach D, Rubinstein M (1989) Soluble cytokine receptors are present in normal human urine. J Exp Med 170: 1409–1414. 2529343 10.1084/jem.170.4.1409 PMC2189483

[74] Peterson PA, Evrin PE, Berggård I (1969) Differentiation of glomerular, tubular, and normal proteinuria: determinations of urinary excretion of ß_2_-microglobulin, albumin, and total protein. J Clin Invest 48: 1189–1198. 4978446 10.1172/JCI106083 PMC322340

[75] Takeyama K, Kitanaka S, Sato T, Kobori M, Yanagisawa J, et al (1997) 25-Hydroxyvitamin D_3_ 1α-hydroxylase and vitamin D synthesis. Science 277: 1827–1830. 9295274 10.1126/science.277.5333.1827

[76] Birn H, Nexø E, Christensen EI, Nielsen R (2003) Diversity in rat tissue accumulation of vitamin B12 supports a distinct role for the kidney in vitamin B12 homeostasis. Nephrol Dial Transplant 18: 1095–1100. 12748340 10.1093/ndt/gfg089

[77] Cavaleros M, Buffenstein R, Ross FP, Pettifor JM (2003) Vitamin D metabolism in a frugivorous nocturnal mammal, the Egyptian fruit bat (*Rousettus aegyptiacus*). Gen Comp Endocrinol 133: 109–117. 12899852 10.1016/s0016-6480(03)00150-3

[78] Dierenfeld ES, Seyjagat J (2000) Plasma fat-soluble vitamin and mineral concentrations in relation to diet in captive pteropodid bats. J Zoo Wildl Med 31: 315–321. 11237137 10.1638/1042-7260(2000)031[0315:PFSVAM]2.0.CO;2

[79] van Tonder SV, Metz J, Green R (1975) Vitamin B12 metabolism in the fruit bat (*Rousettus aegyptiacus*). The induction of vitamin B12 deficiency and its effect on folate levels. Br J Nutr 34: 397–410. 1201264 10.1017/s0007114575000463

[80] Homma K, Yoshimura M, Saito J, Ikebe R, Ikebe M (2001) The core of the motor domain determines the direction of myosin movement. Nature 412: 831–834. 11518969 10.1038/35090597

[81] Hertzano R, Shalit E, Rzadzinska AK, Dror AA, Song L, et al (2008) A Myo6 mutation destroys coordination between the myosin heads, revealing new functions of myosin VI in the stereocilia of mammalian inner ear hair cells. PLoS Genet 4: e1000207. 18833301 10.1371/journal.pgen.1000207 PMC2543112

[82] Aschenbrenner L, Naccache SN, Hasson T (2004) Uncoated endocytic vesicles require the unconventional myosin, Myo6, for rapid transport through actin barriers. Mol Biol Cell 15: 2253–2263. 15004223 10.1091/mbc.E04-01-0002 PMC404020

[83] Aschenbrenner L, Lee T, Hasson T (2003) Myo6 facilitates the translocation of endocytic vesicles from cell peripheries. Mol Biol Cell 14: 2728–2743. 12857860 10.1091/mbc.E02-11-0767 PMC165672

[84] De La Cruz EM, Ostap EM, Sweeney HL (2001) Kinetic mechanism and regulation of myosin VI. J Biol Chem 276: 32373–32381. 11423557 10.1074/jbc.M104136200

[85] Sweeney HL, Houdusse A (2007) What can myosin VI do in cells? Curr Opin Cell Biol 19: 57–66. 17175153 10.1016/j.ceb.2006.12.005

[86] Betts MJ, Russell RB (2003) Amino acid properties and consequences of substitutions. In: Barnes MR, Gray IC, editors. Bioinformatics for geneticists. West Sussex: Wiley.

[87] Altingham JD, McOwat T, Hammond L (1998) Bats: biology and behavior. New York: Oxford University Press.

[88] Herbst LH (1986) The role of nitrogen from fruit pulp in the nutrition of the frugivorous bat *Carollia perspicillata* . Biotropica 18: 39–44.

[89] Shen B, Han X, Zhang J, Rossiter SJ, Zhang S (2012) Adaptive evolution in the glucose transporter 4 gene *Slc2a4* in Old World fruit bats (Family: Pteropodidae). PLoS One 7: e33197. 22493665 10.1371/journal.pone.0033197 PMC3320886

[90] Liu Y, Xu H, Yuan X, Rossiter SJ, Zhang S (2012) Multiple adaptive losses of alanine-glyoxylate aminotransferase mitochondrial targeting in fruit-eating bats. Mol Biol Evol 29: 1507–1511. 22319153 10.1093/molbev/mss013

