# Supplementary figures and images for: Adaptive Evolution of the Myo6 Gene in Old World Fruit Bats (Family: Pteropodidae)
Source: PLoS One. 2013 Apr 19;8(4):e62307. doi: 10.1371/journal.pone.0062307 (PMC3631194; doi:10.1371/journal.pone.0062307)

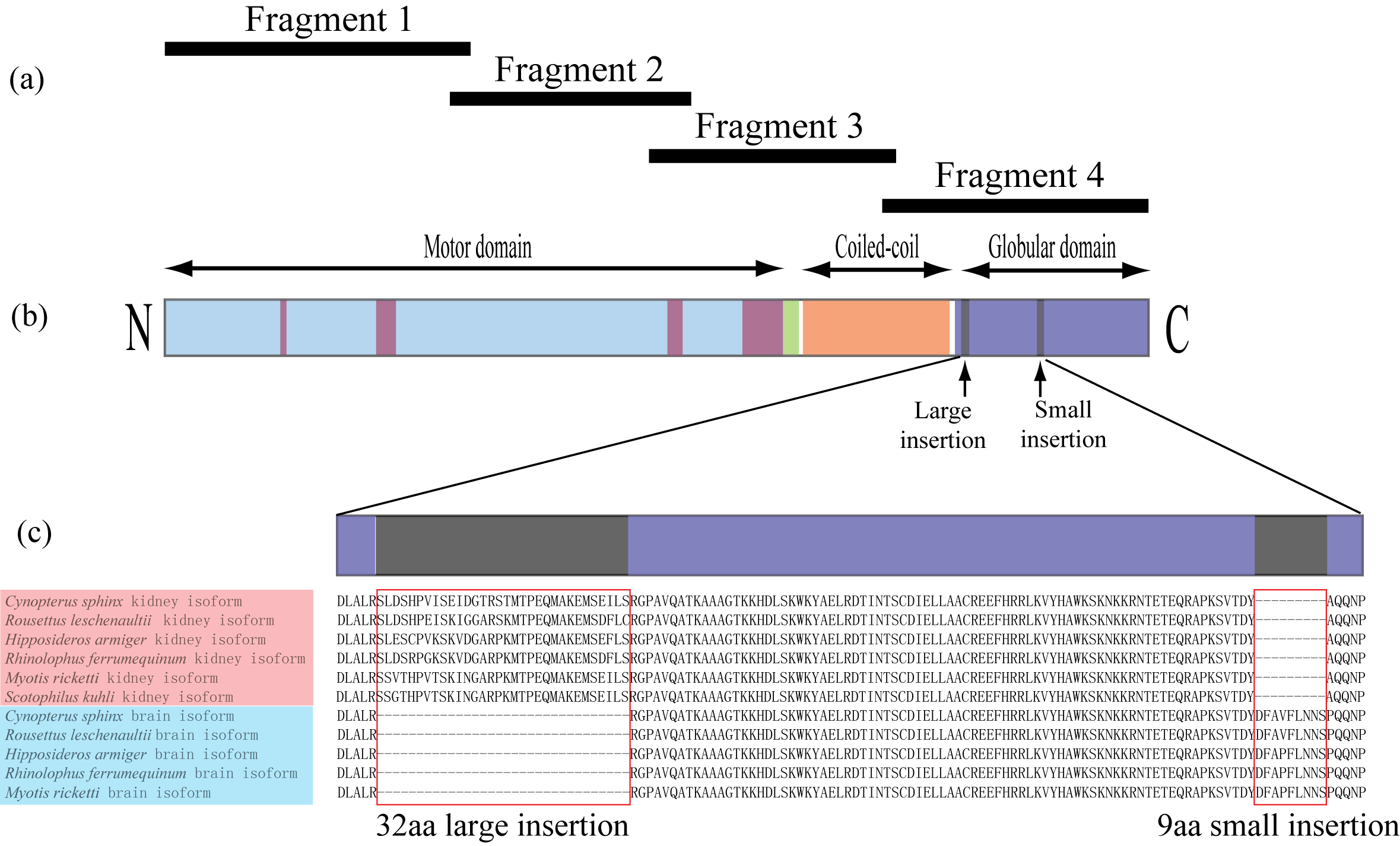

Supplement: Figure S1 — Myosin VI protein structure showing the differences of Myo6 isoforms from brain and kidney. (a) A cartoon illustrating the four overlapped fragments for PCR. The Myo6 coding sequence was divided into four overlapped fragments, for each fragment a pair of primers were designed for amplification. (b) Schematic of myosin VI structure with the large and small insertions in tail domain are shown. (c) Comparison of amino acid sequences of Myo6 isoforms from brain and kidney. The sequences framed are the large insertion (32aa) in the kidney isoform and the small insertion (9aa) in the brain isoform. (TIF) [file pone.0062307.s001.tif]

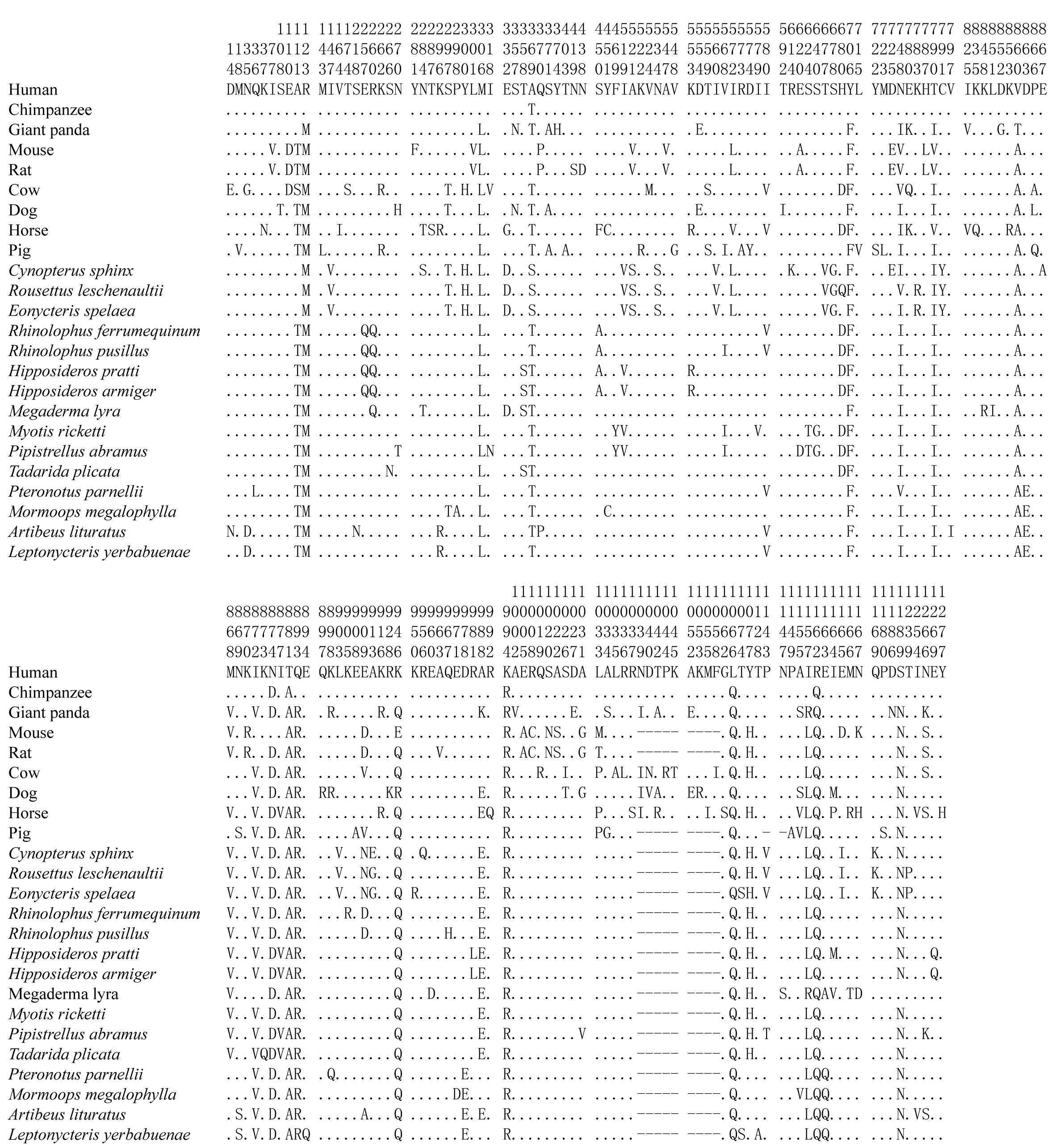

Supplement: Figure S2 — Alignment of the amino acid sequences of the Myo6 gene from 24 mammals (only the variable sites are shown). (TIF) [file pone.0062307.s002.tif]
