# Supplementary material for: Adaptive Evolution of the Myo6 Gene in Old World Fruit Bats (Family: Pteropodidae)
Source: PLoS One. 2013 Apr 19;8(4):e62307. doi: 10.1371/journal.pone.0062307 (PMC3631194; doi:10.1371/journal.pone.0062307)
Supplement: Figure S3 — Alignment of the full amino acid sequences of the Myo6 gene from 24 mammals. Twelve positively selected sites detected in the Old World fruit bats are highlighted by red squares and indicated with asterisks on above of alignment columns. (PDF) [file pone.0062307.s003.pdf]

										1	1111111111	1111111111	1111111111
	1	1111111112	2222222223	3333333334	4444444445	5555555556	6666666667	7777777778	8888888889	9999999990	0000000001	1111111112	2222222223
	1234567890	1234567890	1234567890	1234567890	1234567890	1234567890	1234567890	1234567890	1234567890	1234567890	1234567890	1234567890	1234567890
Human	MEDGKPVWAP	HPTDGFQMGN	IVDIGPDSLT	IEPLNQKGKT	FLALINQVFP	AEEDSKKDVE	DNCSLMYLNE	ATLLHNIKVR	YSKDRIYTYV	ANILIAVNPY	FDIPKIYSSE	AIKSYQGKSL	GTRPPHVFAI
Chimpanzee	MEDGKPVWAP	HPTDGFQMGN	IVDIGPDSLT	IEPLNQKGKT	FLALINQVFP	AEEDSKKDVE	DNCSLMYLNE	ATLLHNIKVR	YSKDRIYTYV	ANILIAVNPY	FDIPKIYSSE	AIKSYQGKSL	GTRPPHVFAI
Panda	MEDGKPVWAP	HPTDGFQMGN	IVDIGPDSLT	IEPLNQKGKT	FLALINQVFP	AEEDSKKDVE	DNCSLMYLNE	ATLLHNIKVR	YSKDRIYTYV	ANILIAVNPY	FDIPKIYSSE	AIKSYQGKSL	GTMPPHVFAI
Mouse	MEDGKPVWAP	HPTDGFQMGN	IVDIGPDSLT	IEPLNQKGKT	FLALINQVFP	AEEDSKKDVE	DNCSLMYLNE	ATLLHNVKVR	YSKDRIYTYV	ANILIAVNPY	FDIPKIYSSD	TIKSYQGKSL	GTMPPHVFAI
Rat	MEDGKPVWAP	HPTDGFQMGN	IVDIGPDSLT	IEPLNQKGKT	FLALINQVFP	AEEDSKKDVE	DNCSLMYLNE	ATLLHNVKVR	YSKDRIYTYV	ANILIAVNPY	FDIPKIYSSD	TIKSYQGKSL	GTMPPHVFAI
Cow	MEDGRPVWAP	HPTEGFQMGN	IVDIGPDSLT	IEPLGQKGKT	FLALINQVFP	AEEDSKKDVE	DNCSLMYLNE	ATLLHNIKVR	YSKDRIYTYV	ANILIAVNPY	FDIPKIYSSD	SIKSYQGKSL	GTMPPHVFAI
Dog	MEDGKPVWAP	HPTDGFQMGN	IVDIGPDSLT	IEPLNQKGKT	FLALINQVFP	AEEDSKKDVE	DNCSLMYLNE	ATLLHNIKVR	YSKDRIYTYV	ANILIAVNPY	FDIPKIYTSE	TIKSYQGKSL	GTMPPHVFAI
Horse	MEDGKPVWAP	HPTDGFQMGN	IVDIGPDSLT	IEPLNQNGKT	FLALINQVFP	AEEDSKKDVE	DNCSLMYLNE	ATLLHNIKVR	YSKDRIYTYV	ANILIAVNPY	FDIPKIYSSE	TIKSYQGKSL	GTMPPHVFAI
Pig	MEDGKPVWAP	HPTDGFQVGN	IVDIGPDSLT	IEPLNQKGKT	FLALINQVFP	AEEDSKKDVE	DNCSLMYLNE	ATLLHNIKVR	YSKDRIYTYV	ANILIAVNPY	FDIPKIYSSE	TIKSYQGKSL	GTMPPHVFAI
Cynopterus sphinx	MEDGKPVWAP	HPTDGFQMGN	IVDIGPDSLT	IEPLNQKGKT	FLALINQVFP	AEEDSKKDVE	DNCSLMYLNE	ATLLHNIKVR	YSKDRIYTYV	ANILIAVNPY	FDIPKIYSSE	AIKSYQGKSL	GTMPPHVFAI
Rousettus leschenaultii	MEDGKPVWAP	HPTDGFQMGN	IVDIGPDSLT	IEPLNQKGKT	FLALINQVFP	AEEDSKKDVE	DNCSLMYLNE	ATLLHNIKVR	YSKDRIYTYV	ANILIAVNPY	FDIPKIYSSE	AIKSYQGKSL	GTMPPHVFAI
Eonycteris spelaea	MEDGKPVWAP	HPTDGFQMGN	IVDIGPDSLT	IEPLNQKGKT	FLALINQVFP	AEEDSKKDVE	DNCSLMYLNE	ATLLHNIKVR	YSKDRIYTYV	ANILIAVNPY	FDIPKIYSSE	AIKSYQGKSL	GTMPPHVFAI
Rhinolophus ferrumequinum	MEDGKPVWAP	HPTDGFQMGN	IVDIGPDSLT	IEPLNQKGKT	FLALINQVFP	AEEDSKKDVE	DNCSLMYLNE	ATLLHNIKVR	YSKDRIYTYV	ANILIAVNPY	FDIPKIYSSE	TIKSYQGKSL	GTMPPHVFAI
Rhinolophus pusillus	MEDGKPVWAP	HPTDGFQMGN	IVDIGPDSLT	IEPLNQKGKT	FLALINQVFP	AEEDSKKDVE	DNCSLMYLNE	ATLLHNIKVR	YSKDRIYTYV	ANILIAVNPY	FDIPKIYSSE	TIKSYQGKSL	GTMPPHVFAI
Hipposideros pratti	MEDGKPVWAP	HPTDGFQMGN	IVDIGPDSLT	IEPLNQKGKT	FLALINQVFP	AEEDSKKDVE	DNCSLMYLNE	ATLLHNIKVR	YSKDRIYTYV	ANILIAVNPY	FDIPKIYSSE	TIKSYQGKSL	GTMPPHVFAI
Hipposideros armiger	MEDGKPVWAP	HPTDGFQMGN	IVDIGPDSLT	IEPLNQKGKT	FLALINQVFP	AEEDSKKDVE	DNCSLMYLNE	ATLLHNIKVR	YSKDRIYTYV	ANILIAVNPY	FDIPKIYSSE	TIKSYQGKSL	GTMPPHVFAI
Megaderma lyra	MEDGKPVWAP	HPTDGFQMGN	IVDIGPDSLT	IEPLNQKGKT	FLALINQVFP	AEEDSKKDVE	DNCSLMYLNE	ATLLHNIKVR	YSKDRIYTYV	ANILIAVNPY	FDIPKIYSSE	TIKSYQGKSL	GTMPPHVFAI
Myotis ricketti	MEDGKPVWAP	HPTDGFQMGN	IVDIGPDSLT	IEPLNQKGKT	FLALINQVFP	AEEDSKKDVE	DNCSLMYLNE	ATLLHNIKVR	YSKDRIYTYV	ANILIAVNPY	FDIPKIYSSE	TIKSYQGKSL	GTMPPHVFAI
Pipistrellus abramus	MEDGKPVWAP	HPTDGFQMGN	IVDIGPDSLT	IEPLNQKGKT	FLALINQVFP	AEEDSKKDVE	DNCSLMYLNE	ATLLHNIKVR	YSKDRIYTYV	ANILIAVNPY	FDIPKIYSSE	TIKSYQGKSL	GTMPPHVFAI
Tadarida plicata	MEDGKPVWAP	HPTDGFQMGN	IVDIGPDSLT	IEPLNQKGKT	FLALINQVFP	AEEDSKKDVE	DNCSLMYLNE	ATLLHNIKVR	YSKDRIYTYV	ANILIAVNPY	FDIPKIYSSE	TIKSYQGKSL	GTMPPHVFAI
Pteronotus parnellii	MEDGKPVWAP	HPTDGFQMGN	IVDIGPDSLT	IEPLNLKGKT	FLALINQVFP	AEEDSKKDVE	DNCSLMYLNE	ATLLHNIKVR	YSKDRIYTYV	ANILIAVNPY	FDIPKIYSSE	TIKSYQGKSL	GTMPPHVFAI
Mormoops megalophylla	MEDGKPVWAP	HPTDGFQMGN	IVDIGPDSLT	IEPLNQKGKT	FLALINQVFP	AEEDSKKDVE	DNCSLMYLNE	ATLLHNIKVR	YSKDRIYTYV	ANILIAVNPY	FDIPKIYSSE	TIKSYQGKSL	GTMPPHVFAI
Artibeus lituratus	MEDGKPVWAP	HPTNGFQMGN	IVDIGPDSLT	IEPLDQKGKT	FLALINQVFP	AEEDSKKDVE	DNCSLMYLNE	ATLLHNIKVR	YSKDRIYTYV	ANILIAVNPY	FDIPKIYSSE	TIKSYQGKSL	GTMPPHVFAI
Leptonycteris yerbabuenae	MEDGKPVWAP	HPTDGFQMGN	IVDIGPDSLT	IEPLDQKGKT	FLALINQVFP	AEEDSKKDVE	DNCSLMYLNE	ATLLHNIKVR	YSKDRIYTYV	ANILIAVNPY	FDIPKIYSSE	TIKSYQGKSL	GTMPPHVFAI

	1111111111	1111111111	1111111111	1111111111	1111111111	1111111111	1111111112	2222222222	2222222222	2222222222	2222222222	2222222222	2222222222
	3333333334	4444444445	5555555556	6666666667	7777777778	8888888889	9999999990	0000000001	1111111112	2222222223	3333333334	4444444445	5555555556
	1234567890	1234567890	1234567890	1234567890	1234567890	1234567890	1234567890	1234567890	1234567890	1234567890	1234567890	1234567890	1234567890
Human	ADKAFRDMKV	LKMSQSIIVS	GESGAGKTEN	TKFVLRYLTE	SYGTGQDIDD	RIVEANPLLE	AFGNAKTVRN	NNSSRFGKFV	EIHFNEKSSV	VGGFVSHYLL	EKSRICVQGK	EERNYHIFYR	LCAGASEDIR
Chimpanzee	ADKAFRDMKV	LKMSQSIIVS	GESGAGKTEN	TKFVLRYLTE	SYGTGQDIDD	RIVEANPLLE	AFGNAKTVRN	NNSSRFGKFV	EIHFNEKSSV	VGGFVSHYLL	EKSRICVQGK	EERNYHIFYR	LCAGASEDIR
Panda	ADKAFRDMKV	LKMSQSIIVS	GESGAGKTEN	TKFVLRYLTE	SYGTGQDIDD	RIVEANPLLE	AFGNAKTVRN	NNSSRFGKFV	EIHFNEKSSV	VGGFVSHYLL	EKSRICVQGK	EERNYHIFYR	LCAGASEDIR
Mouse	ADKAFRDMKV	LKMSQSIIVS	GESGAGKTEN	TKFVLRYLTE	SYGTGQDIDD	RIVEANPLLE	AFGNAKTVRN	NNSSRFGKFV	EIHFNEKSSV	VGGFVSHYLL	EKSRICVQGK	EERNYHIFYR	LCAGASEDIR
Rat	ADKAFRDMKV	LKMSQSIIVS	GESGAGKTEN	TKFVLRYLTE	SYGTGQDIDD	RIVEANPLLE	AFGNAKTVRN	NNSSRFGKFV	EIHFNEKSSV	VGGFVSHYLL	EKSRICVQGK	EERNYHIFYR	LCAGASEDIR
Cow	ADKAFRDMKV	LKMSQSIIVS	GESGAGKTEN	TKFVLRYLTE	SYGSGQDIDD	RIVEANPLLE	AFGNAKTVRN	NNSSRFGKFV	EIHFNEKSSV	VGGFVSHYLL	EKSRICVQGK	EERNYHIFYR	LCAGASEDIR
Dog	ADKAFRDMKV	LKMSQSIIVS	GESGAGKTEN	TKFVLRYLTE	SYGTGQDIDD	RIVEANPLLE	AFGNAKTVRN	NNSSRFGKFV	EIHFNEKSSV	VGGFVSHYLL	EKSRICVQGK	EERNYHIFYR	LCAGASEDIR
Horse	ADKAFRDMKV	LKMSQSIIVS	GESGAGKTEN	TKFILRYLTE	SYGTGQDIDD	RIVEANPLLE	AFGNAKTVRN	NNSSRFGKFV	EIHFNEKSSV	VGGFVSHYLL	EKSRICVQGK	EERNYHIFYR	LCAGASEDIR
Pig	ADKAFRDMKV	LKLSQSIIVS	GESGAGKTEN	TKFVLRYLTE	SYGTGQDIDD	RIVEANPLLE	AFGNAKTVRN	NNSSRFGKFV	EIHFNEKSSV	VGGFVSHYLL	EKSRICVQGK	EERNYHIFYR	LCAGASEDIR
Cynopterus sphinx	ADKAFRDMKV	LKMSQSVIVS	GESGAGKTEN	TKFVLRYLTE	SYGTGQDIDD	RIVEANPLLE	AFGNAKTVRN	NNSSRFGKFV	EIHFNEKSSV	VGGFVSHYLL	EKSRICVQGK	EERNYHIFYR	LCAGASEDIR
Rousettus leschenaultii	ADKAFRDMKV	LKMSQSVIVS	GESGAGKTEN	TKFVLRYLTE	SYGTGQDIDD	RIVEANPLLE	AFGNAKTVRN	NNSSRFGKFV	EIHFNEKSSV	VGGFVSHYLL	EKSRICVQGK	EERNYHIFYR	LCAGASEDIR
Eonycteris spelaea	ADKAFRDMKV	LKMSQSVIVS	GESGAGKTEN	TKFVLRYLTE	SYGTGQDIDD	RIVEANPLLE	AFGNAKTVRN	NNSSRFGKFV	EIHFNEKSSV	VGGFVSHYLL	EKSRICVQGK	EERNYHIFYR	LCAGASEDIR
Rhinolophus ferrumequinum	ADKAFRDMKV	LKMSQSIIVS	GESGAGKTEN	TKFVLRYLTE	SYGTGQDIDD	RIVEANPLLE	AFGNAKTVRN	NNSSRFGKFV	EIHFNEKSSV	VGGFVSHYLL	EKSRICVQGK	EERNYHIFYR	LCAGASQDIQ
Rhinolophus pusillus	ADKAFRDMKV	LKMSQSIIVS	GESGAGKTEN	TKFVLRYLTE	SYGTGQDIDD	RIVEANPLLE	AFGNAKTVRN	NNSSRFGKFV	EIHFNEKSSV	VGGFVSHYLL	EKSRICVQGK	EERNYHIFYR	LCAGASQDIQ
Hipposideros pratti	ADKAFRDMKV	LKMSQSIIVS	GESGAGKTEN	TKFVLRYLTE	SYGTGQDIDD	RIVEANPLLE	AFGNAKTVRN	NNSSRFGKFV	EIHFNEKSSV	VGGFVSHYLL	EKSRICVQGK	EERNYHIFYR	LCAGASQDIQ
Hipposideros armiger	ADKAFRDMKV	LKMSQSIIVS	GESGAGKTEN	TKFVLRYLTE	SYGTGQDIDD	RIVEANPLLE	AFGNAKTVRN	NNSSRFGKFV	EIHFNEKSSV	VGGFVSHYLL	EKSRICVQGK	EERNYHIFYR	LCAGASQDIQ
Megaderma lyra	ADKAFRDMKV	LKMSQSIIVS	GESGAGKTEN	TKFVLRYLTE	SYGTGQDIDD	RIVEANPLLE	AFGNAKTVRN	NNSSRFGKFV	EIHFNEKSSV	VGGFVSHYLL	EKSRICVQGK	EERNYHIFYR	LCAGASEDIQ
Myotis ricketti	ADKAFRDMKV	LKMSQSIIVS	GESGAGKTEN	TKFVLRYLTE	SYGTGQDIDD	RIVEANPLLE	AFGNAKTVRN	NNSSRFGKFV	EIHFNEKSSV	VGGFVSHYLL	EKSRICVQGK	EERNYHIFYR	LCAGASEDIR
Pipistrellus abramus	ADKAFRDMKV	LKMSQSIIVS	GESGAGKTEN	TKFVLRYLTE	SYGTGQDIDD	RIVEANPLLE	AFGNAKTVRN	NNSSRFGKFV	EIHFNEKSSV	VGGFVSHYLL	EKSRICVQGK	EERNYHIFYR	LCAGASEDIR
Tadarida plicata	ADKAFRDMKV	LKMSQSIIVS	GESGAGKTEN	TKFVLRYLTE	SYGTGQDIDD	RIVEANPLLE	AFGNAKTVRN	NNSSRFGKFV	EIHFNEKSSV	VGGFVSHYLL	EKSRICVQGK	EERNYHIFYR	LCAGASEDIR
Pteronotus parnellii	ADKAFRDMKV	LKMSQSIIVS	GESGAGKTEN	TKFVLRYLTE	SYGTGQDIDD	RIVEANPLLE	AFGNAKTVRN	NNSSRFGKFV	EIHFNEKSSV	VGGFVSHYLL	EKSRICVQGK	EERNYHIFYR	LCAGASEDIR
Mormoops megalophylla	ADKAFRDMKV	LKMSQSIIVS	GESGAGKTEN	TKFVLRYLTE	SYGTGQDIDD	RIVEANPLLE	AFGNAKTVRN	NNSSRFGKFV	EIHFNEKSSV	VGGFVSHYLL	EKSRICVQGK	EERNYHIFYR	LCAGASEDIR
Artibeus lituratus	ADKAFRDMKV	LKMSQSIIVS	GESGAGKTEN	TKFVLRYLTE	SYGTGQDIDD	RIVEANPLLE	AFGNAKTVRN	NNSSRFGKFV	EIHFNEKNSV	VGGFVSHYLL	EKSRICVQGK	EERNYHIFYR	LCAGASEDIR
Leptonycteris yerbabuenae	ADKAFRDMKV	LKMSQSIIVS	GESGAGKTEN	TKFVLRYLTE	SYGTGQDIDD	RIVEANPLLE	AFGNAKTVRN	NNSSRFGKFV	EIHFNEKSSV	VGGFVSHYLL	EKSRICVQGK	EERNYHIFYR	LCAGASEDIR

	2222222222	2222222222	2222222222	2222222223	3333333333	3333333333	3333333333	3333333333	3333333333	3333333333	3333333333	3333333333	3333333333
	6666666667	7777777778	8888888889	9999999990	0000000001	1111111112	2222222223	3333333334	4444444445	5555555556	6666666667	7777777778	8888888889
	1234567890	1234567890	1234567890	1234567890	1234567890	1234567890	1234567890	1234567890	1234567890	1234567890	1234567890	1234567890	1234567890
Human	EKLHLSSPDN	FRYLNRGCTR	YFANKETDKQ	ILQNRKSPEY	LKAGSMKDPL	LDDHGDFIRM	CTAMKKIGLD	DEEKLDLFRV	VAGVLHLGNI	DFEEAGSTSG	GCNLKNKSAQ	SLEYCAE-LL	GLDQDDLRVS
Chimpanzee	EKLHLSSPDN	FRYLNRGCTR	YFANKETDKQ	ILQNRKSPEY	LKAGSMKDPL	LDDHGDFIRM	CTAMKKIGLD	DEEKLDLFRV	VAGVLHLGNI	DFEEAGSTSG	GCNLKNKSTQ	SLEYCAE-LL	GLDQDDLRVS
Panda	EKLHLSSPDN	FRYLNRGCTR	YFANKETDKQ	ILQNRKSPEY	LKAGSLKDPL	LDDHGDFIRM	CTAMKKIGLD	DEEKLDLFRV	VAGVLHLGNI	DFEEAGNTSG	GCNLKNKSTQ	ALEHCAE-LL	GLDQDDLRVS
Mouse	EKLHLSSPDN	FRYLNRGCTR	FFANKETDKQ	ILQNRKSPEY	VKAGSLKDPL	LDDHGDFIRM	CTAMKKIGLD	DEEKLDLFRV	VAGVLHLGNI	DFEEAGSTSG	GCNLKNKSAP	SLEYCAE-LL	GLDQDDLRVS
Rat	EKLHLSSPDN	FRYLNRGCTR	YFANKETDKQ	ILQNRKSPEY	VKAGSLKDPL	LDDHGDFIRM	CTAMKKIGLD	DEEKLDLFRV	VAGVLHLGNI	DFEEAGSTSG	GCNLKNKSAP	SLEYCAE-LL	GLDQDDLRVS
Cow	ERLHLSSPDN	FRYLNRGCTR	YFANKETDKQ	ILQNRKTPEH	LKAGSLKDPL	LDDHGDFVRM	CTAMKKIGLD	DEEKLDLFRV	VAGVLHLGNI	DFEEAGSTSG	GCNLKNKSTQ	SLEYCAE-LL	GLDQDDLRVS
Dog	EKLHLSSPDH	FRYLNRGCTR	YFANKETDKQ	ILQNRKTPEY	LKAGSLKDPL	LDDHGDFIRM	CTAMKKIGLD	DEEKLDLFRV	VAGVLHLGNI	DFEEAGNTSG	GCNLKNKSTQ	ALEYCAE-LL	GLDQDDLRVS
Horse	EKLHLSSPDN	FRYLNRGCTR	YFATKESDKQ	ILQNRRSPEY	LKAGSLKDPL	LDDHGDFIRM	CTAMKKIGLD	DGEKLDLFRV	VAGVLHLGNI	DFEEAGSTSG	GCNLKNKSTQ	SLEYCAE-LL	GLDQDDLRVS
Pig	ERLHLSSPDN	FRYLNRGCTR	YFANKETDKQ	ILQNRKSPEY	LKAGSLKDPL	LDDHGDFIRM	CTAMKKIGLD	DEEKLDLFRV	VAGVLHLGNI	DFEEAGSTSG	GCNLKNKSTQ	ALEYCAEKLL	GLDQDDLRVS
Cynopterus sphinx	EKLHLSSPDN	FRYLNRGCTR	YFASKETDKQ	ILQNRKTPEH	LKAGSLKDPL	LDDHGDFIRM	CTAMKKIGLD	DDEKLDLFRV	VAGVLHLGNI	DFEEAGSTSG	GCNLKNKSSQ	SLEYCAE-LL	GLDQDDLRVS
Rousettus leschenaultii	EKLHLSSPDN	FRYLNRGCTR	YFANKETDKQ	ILQNRKTPEH	LKAGSLKDPL	LDDHGDFIRM	CTAMKKIGLD	DDEKLDLFRV	VAGVLHLGNI	DFEEAGSTSG	GCNLKNKSSQ	SLEYCAE-LL	GLDQDDLRVS
Eonycteris spelaea	EKLHLSSPDN	FRYLNRGCTR	YFANKETDKQ	ILQNRKTPEH	LKAGSLKDPL	LDDHGDFIRM	CTAMKKIGLD	DDEKLDLFRV	VAGVLHLGNI	DFEEAGSTSG	GCNLKNKSSQ	SLEYCAE-LL	GLDQDDLRVS
Rhinolophus ferrumequinum	EKLHLSSPDN	FRYLNRGCTR	YFANKETDKQ	ILQNRKSPEY	LKAGSLKDPL	LDDHGDFIRM	CTAMKKIGLD	DEEKLDLFRV	VAGVLHLGNI	DFEEAGSTSG	GCNLKNKSTQ	SLEYCAE-LL	GLDQDDLRVS
Rhinolophus pusillus	EKLHLSSPDN	FRYLNRGCTR	YFANKETDKQ	ILQNRKSPEY	LKAGSLKDPL	LDDHGDFIRM	CTAMKKIGLD	DEEKLDLFRV	VAGVLHLGNI	DFEEAGSTSG	GCNLKNKSTQ	SLEYCAE-LL	GLDQDDLRVS
Hipposideros pratti	EKLHLSSPDN	FRYLNRGCTR	YFANKETDKQ	ILQNRKSPEY	LKAGSLKDPL	LDDHGDFIRM	CTAMKKIGLD	DEEKLDLFRV	VAGVLHLGNI	DFEEAGSSSG	GCNLKNKSTQ	SLEYCAE-LL	GLDQDDLRVS
Hipposideros armiger	EKLHLSSPDN	FRYLNRGCTR	YFANKETDKQ	ILQNRKSPEY	LKAGSLKDPL	LDDHGDFIRM	CTAMKKIGLD	DEEKLDLFRV	VAGVLHLGNI	DFEEAGSSSG	GCNLKNKSTQ	SLEYCAE-LL	GLDQDDLRVS
Megaderma lyra	EKLHLSSPDN	FRYLNRGCTR	YFATKETDKQ	ILQNRKSPEY	LKAGSLKDPL	LDDHGDFIRM	CTAMKKIGLD	DDEKLDLFRV	VAGVLHLGNI	DFEEAGSSSG	GCNLKNKSTQ	SLEYCAE-LL	GLDQDDLRVS
Myotis ricketti	EKLHLSSPDN	FRYLNRGCTR	YFANKETDKQ	ILQNRKSPEY	LKAGSLKDPL	LDDHGDFIRM	CTAMKKIGLD	DEEKLDLFRV	VAGVLHLGNI	DFEEAGSTSG	GCNLKNKSTQ	SLEYCAE-LL	GLDQDDLRVS
Pipistrellus abramus	EKLHLSSPDT	FRYLNRGCTR	YFANKETDKQ	ILQNRKSPEY	LKAGSLKDPL	LDDHGDFNRM	CTAMKKIGLD	DEEKLDLFRV	VAGVLHLGNI	DFEEAGSTSG	GCNLKNKSTQ	SLEYCAE-LL	GLDQDDLRVS
Tadarida plicata	EKLHLNSPDN	FRYLNRGCTR	YFANKETDKQ	ILQNRKSPEY	LKAGSLKDPL	LDDHGDFIRM	CTAMKKIGLD	DEEKLDLFRV	VAGVLHLGNI	DFEEAGSSSG	GCNLKNKSTQ	SLEYCAE-LL	GLDQDDLRVS
Pteronotus parnellii	EKLHLSSPDN	FRYLNRGCTR	YFANKETDKQ	ILQNRKSPEY	LKAGSLKDPL	LDDHGDFIRM	CTAMKKIGLD	DEEKLDLFRV	VAGVLHLGNI	DFEEAGSTSG	GCNLKNKSTQ	SLEYCAE-LL	GLDQDDLRVS
Mormoops megalophylla	EKLHLSSPDN	FRYLNRGCTR	YFANKETDKQ	ILQNRKTAEY	LKAGSLKDPL	LDDHGDFIRM	CTAMKKIGLD	DEEKLDLFRV	VAGVLHLGNI	DFEEAGSTSG	GCNLKNKSTQ	SLEYCAE-LL	GLDQDDLRVS
Artibeus lituratus	EKLHLSSPDN	FRYLNRGCTR	YFANKETDKQ	ILQNRRSPEY	LKAGSLKDPL	LDDHGDFIRM	CTAMKKIGLD	DEEKLDLFRV	VAGVLHLGNI	DFEEAGSTSG	GCNLKNKSTP	SLEYCAE-LL	GLDQDDLRVS
Leptonycteris yerbabuenae	EKLHLSSPDN	FRYLNRGCTR	YFANKETDKQ	ILQNRRSPEY	LKAGSLKDPL	LDDHGDFIRM	CTAMKKIGLD	DEEKLDLFRV	VAGVLHLGNI	DFEEAGSTSG	GCNLKNKSTQ	SLEYCAE-LL	GLDQDDLRVS

	3333333334	4444444444	4444444444	4444444444	4444444444	4444444444	4444444444	4444444444	4444444444	4444444444	4444444445	5555555555	5555555555
	9999999990	0000000001	1111111112	2222222223	3333333334	4444444445	5555555556	6666666667	7777777778	8888888889	9999999990	0000000001	1111111112
	1234567890	1234567890	1234567890	1234567890	1234567890	1234567890	1234567890	1234567890	1234567890	1234567890	1234567890	1234567890	1234567890
Human	LTTRVMLTTA	GGTKGTVIKV	PLKVEQANNA	RDALAKTVYS	HLFDHVVNRV	NQCFPFETSS	YFIGVLDIAG	FEYFEHNSFE	QFCINYCNEK	LQQFFNERIL	KEEQELYQKE	GLGVNEVHYV	DNQDCIDLIE
Chimpanzee	LTTRVMLTTA	GGTKGTVIKV	PLKVEQANNA	RDALAKTVYS	HLFDHVVNRV	NQCFPFETSS	YFIGVLDIAG	FEYFEHNSFE	QFCINYCNEK	LQQFFNERIL	KEEQELYQKE	GLGVNEVHYV	DNQDCIDLIE
Panda	LTTRVMLTTA	GGTKGTVIKV	PLKVEQANNA	RDALAKTVYS	HLFDHVVNRV	NQCFPFETSS	YFIGVLDIAG	FEYFEHNSFE	QFCINYCNEK	LQQFFNERIL	KEEQELYQKE	GLGVNEVHYV	DNQDCIDLIE
Mouse	LTTRVMLTTA	GGTKGTVIKV	PLKVEQANNA	RDALAKTVYS	HLFDHVVNRV	NQCFPFETSS	YFIGVLDIAG	FEYFEHNSFE	QFCINYCNEK	LQQFFNERIL	KEEQELYQKE	GLGVNEVHYV	DNQDCIDLIE
Rat	LTTRVMLTTA	GGTKGTVIKV	PLKVEQANSA	RDALAKTVYS	HLFDHVVDRV	NQCFPFETSS	YFIGVLDIAG	FEYFEHNSFE	QFCINYCNEK	LQQFFNERIL	KEEQELYQKE	GLGVNEVHYV	DNQDCIDLIE
Cow	LTTRVMLTTA	GGTKGTVIKV	PLKVEQANNA	RDALAKTVYS	HLFDHVVNRV	NQCFPFETSS	YFIGVLDIAG	FEYFEHNSFE	QFCINYCNEK	LQQFFNERIL	KEEQELYQKE	GLGVNEVHYV	DNQDCIDLIE
Dog	LTTRVMLTTA	GGTKGTVIKV	PLKVEQANNA	RDALAKTVYS	HLFDHVVNRV	NQCFPFETSS	YFIGVLDIAG	FEYFEHNSFE	QFCINYCNEK	LQQFFNERIL	KEEQELYQKE	GLGVNEVHYV	DNQDCIDLIE
Horse	LTTRVMLTTA	GGTKGTVIKV	PLKVEQANNA	RDALAKTVYS	HLFDHVVNRV	NQCFPFETSF	CFIGVLDIAG	FEYFEHNSFE	QFCINYCNEK	LQQFFNERIL	KEEQELYQKE	GLGVNEVHYV	DNQDCIDLIE
Pig	LTTRVMLTTA	GGAKGTVIKV	PLKVEQANNA	RDALAKTVYS	HLFDHVVNRV	NQCFPFETSS	YFIGVLDIAG	FEYFEHNSFE	QFCINYCNEK	LQQFFNERIL	KEEQELYQKE	GLGVNEVHYV	DNQDCIDLIE
Cynopterus sphinx	LTTRVMLTTA	GGTKGTVIKV	PLKVEQANNA	RDALAKTVYS	HLFDHVVNRV	NQCFPFETSS	YFIGVLDIAG	FEYFEHNSFE	QFCINYCNEK	LQQFFNERIL	KEEQELYQKE	GLGVNEVHYV	DNQDCIDLVE
Rousettus leschenaultii	LTTRVMLTTA	GGTKGTVIKV	PLKVEQANNA	RDALAKTVYS	HLFDHVVNRV	NQCFPFETSS	YFIGVLDIAG	FEYFEHNSFE	QFCINYCNEK	LQQFFNERIL	KEEQELYQKE	GLGVNEVHYV	DNQDCIDLVE
Eonycteris spelaea	LTTRVMLTTA	GGTKGTVIKV	PLKVEQANNA	RDALAKTVYS	HLFDHVVNRV	NQCFPFETSS	YFIGVLDIAG	FEYFEHNSFE	QFCINYCNEK	LQQFFNERIL	KEEQELYQKE	GLGVNEVHYV	DNQDCIDLVE
Rhinolophus ferrumequinum	LTTRVMLTTA	GGTKGTVIKV	PLKVEQANNA	RDALAKTVYS	HLFDHVVNRV	NQCFPFETSA	YFIGVLDIAG	FEYFEHNSFE	QFCINYCNEK	LQQFFNERIL	KEEQELYQKE	GLGVNEVHYV	DNQDCIDLIE
Rhinolophus pusillus	LTTRVMLTTA	GGTKGTVIKV	PLKVEQANNA	RDALAKTVYS	HLFDHVVNRV	NQCFPFETSA	YFIGVLDIAG	FEYFEHNSFE	QFCINYCNEK	LQQFFNERIL	KEEQELYQKE	GLGVNEVHYV	DNQDCIDLIE
Hipposideros pratti	LTTRVMLTTA	GGTKGTVIKV	PLKVEQANNA	RDALAKTVYS	HLFDHVVNRV	NQCFPFETSA	YFIGVLDIAG	FEYFEHNSFE	QFCINYCNEK	LQQFFNERIL	KEEQELYQKE	GLGVNEVHYV	DNQDCIDLVE
Hipposideros armiger	LTTRVMLTTA	GGTKGTVIKV	PLKVEQANNA	RDALAKTVYS	HLFDHVVNRV	NQCFPFETSA	YFIGVLDIAG	FEYFEHNSFE	QFCINYCNEK	LQQFFNERIL	KEEQELYQKE	GLGVNEVHYV	DNQDCIDLVE
Megaderma lyra	LTTRVMLTTA	GGTKGTVIKV	PLKVEQANNA	RDALAKTVYS	HLFDHVVNRV	NQCFPFETSS	YFIGVLDIAG	FEYFEHNSFE	QFCINYCNEK	LQQFFNERIL	KEEQELYQKE	GLGVNEVHYV	DNQDCIDLIE
Myotis ricketti	LTTRVMLTTA	GGTKGTVIKV	PLKVEQANNA	RDALAKTVYS	HLFDHVVNRV	NQCFPFETSS	YFIGVLDIAG	FEYFEHNSYE	QFCINYCNEK	LQQFFNERIL	KEEQELYQKE	GLGVNEVHYV	DNQDCIDLVE
Pipistrellus abramus	LTTRVMLTTA	GGTKGTVIKV	PLKVEQANNA	RDALAKTVYS	HLFDHVVNRV	NQCFPFETSS	YFIGVLDIAG	FEYFEHNSYE	QFCINYCNEK	LQQFFNERIL	KEEQELYQKE	GLGVNEVHYV	DNQDCIDLVE
Tadarida plicata	LTTRVMLTTA	GGTKGTVIKV	PLKVEQANNA	RDALAKTVYS	HLFDHVVNRV	NQCFPFETSS	YFIGVLDIAG	FEYFEHNSFE	QFCINYCNEK	LQQFFNERIL	KEEQELYQKE	GLGVNEVHYV	DNQDCIDLIE
Pteronotus parnellii	LTTRVMLTTA	GGTKGTVIKV	PLKVEQANNA	RDALAKTVYS	HLFDHVVNRV	NQCFPFETSS	YFIGVLDIAG	FEYFEHNSFE	QFCINYCNEK	LQQFFNERIL	KEEQELYQKE	GLGVNEVHYV	DNQDCIDLIE
Mormoops megalophylla	LTTRVMLTTA	GGTKGTVIKV	PLKVEQANNA	RDALAKTVYS	HLFDHVVNRV	NQCFPFETSS	CFIGVLDIAG	FEYFEHNSFE	QFCINYCNEK	LQQFFNERIL	KEEQELYQKE	GLGVNEVHYV	DNQDCIDLIE
Artibeus lituratus	LTTRVMLTTA	GGTKGTVIKV	PLKVEQANNA	RDALAKTVYS	HLFDHVVNRV	NQCFPFETSS	YFIGVLDIAG	FEYFEHNSFE	QFCINYCNEK	LQQFFNERIL	KEEQELYQKE	GLGVNEVHYV	DNQDCIDLIE
Leptonycteris yerbabuenae	LTTRVMLTTA	GGTKGTVIKV	PLKVEQANNA	RDALAKTVYS	HLFDHVVNRV	NQCFPFETSS	YFIGVLDIAG	FEYFEHNSFE	QFCINYCNEK	LQQFFNERIL	KEEQELYQKE	GLGVNEVHYV	DNQDCIDLIE

	*	*		*									
	5555555555	5555555555	5555555555	5555555555	5555555555	5555555555	5555555555	5555555556	6666666666	6666666666	6666666666	6666666666	6666666666
	2222222223	3333333334	4444444445	5555555556	6666666667	7777777778	8888888889	9999999990	0000000001	1111111112	2222222223	3333333334	4444444445
	1234567890	1234567890	1234567890	1234567890	1234567890	1234567890	1234567890	1234567890	1234567890	1234567890	1234567890	1234567890	1234567890
Human	AKLVGILDIL	DEENRLPQPS	DQHFTSAVHQ	KHKDHFRLTI	PRKSKLAVHR	NIRDDEGFII	RHFAGAVCYE	TTQFVEKNND	ALHMSLESLI	CESRDKFIRE	LFESSTNNNK	DTKQKAGKLS	FISVGNKFKT
Chimpanzee	AKLVGILDIL	DEENRLPQPS	DQHFTSAVHQ	KHKDHFRLTI	PRKSKLAVHR	NIRDDEGFII	RHFAGAVCYE	TTQFVEKNND	ALHMSLESLI	CESRDKFIRE	LFESSTNNNK	DTKQKAGKLS	FISVGNKFKT
Panda	AKLVGILDIL	DEENRLPQPS	DQHFTSAVHQ	KHKEHFRLTI	PRKSKLAVHR	NIRDDEGFII	RHFAGAVCYE	TTQFVEKNND	ALHMSLESLI	CESRDKFIRE	LFESSTNNNK	DTKQKAGKLS	FISVGNKFKT
Mouse	VKLVGILDIL	DEENRLPQPS	DQHFTSVVHQ	KHKDHFRLTI	PRKSKLAVHR	NLRDDEGFII	RHFAGAVCYE	TTQFVEKNND	ALHMSLESLI	CESRDKFIRA	LFESSTNNNK	DTKQKAGKLS	FISVGNKFKT
Rat	VKLVGILDIL	DEENRLPQPS	DQHFTSVVHQ	KHKDHFRLTI	PRKSKLAVHR	NLRDDEGFII	RHFAGAVCYE	TTQFVEKNND	ALHMSLESLI	CESRDKFIRA	LFESSTNNNK	DTKQKAGKLS	FISVGNKFKT
Cow	AKLMGILDIL	DEENRLPQPS	DQHFTSAVHQ	KHKDHFRLSI	PRKSKLAVHR	NIRDDEGFIV	RHFAGAVCYE	TTQFVEKNND	ALHMSLESLI	CESRDKFIRE	LFESSTNNNK	DTKQKAGKLS	FISVGNKFKT
Dog	AKLVGILDIL	DEENRLPQPS	DQHFTSAVHQ	KHKEHFRLTI	PRKSKLAVHR	NIRDDEGFII	RHFAGAVCYE	TIQFVEKNND	ALHMSLESLI	CESRDKFIRE	LFESSTNNNK	DTKQKAGKLS	FISVGNKFKT
Horse	AKLVGILDIL	DEENRLPQPS	DQHFTSAVHQ	KHRDHFRLTI	PRKSKLAVHR	NVRDDEGFIV	RHFAGAVCYE	TTQFVEKNND	ALHMSLESLI	CESRDKFIRE	LFESSTNNNK	DTKQKAGKLS	FISVGNKFKT
Pig	ARLVGILDIL	DEENRLPQPS	DQHFTSAGHQ	KHKDHFRLSI	PRKSKLAIHR	NIAYDEGFII	RHFAGAVCYE	TTQFVEKNND	ALHMSLESLI	CESRDKFIRE	LFESSTNNNK	DTKQKAGKLS	FISVGNKFKT
Cynopterus sphinx	SKLVGILDIL	DEESRLPQPS	DQHFTSAVHQ	KHKDHFRLTV	PRKSKLAVHR	NLRDDEGFII	RHFAGAVCYE	TTQFVEKNND	ALHMSLESLI	CESKDKFIRE	LFESSTNNNK	DTKQKAGKLS	FISVGNKFKT
Rousettus leschenaultii	SKLVGILDIL	DEESRLPQPS	DQHFTSAVHQ	KHKDHFRLTV	PRKSKLAVHR	NLRDDEGFII	RHFAGAVCYE	TTQFVEKNND	ALHMSLESLI	CESRDKFIRE	LFESSTNNNK	DTKQKAGKLS	FISVGNKFKT
Eonycteris spelaea	SKLVGILDIL	DEESRLPQPS	DQHFTSAVHQ	KHKDHFRLTV	PRKSKLAVHR	NLRDDEGFII	RHFAGAVCYE	TTQFVEKNND	ALHMSLESLI	CESRDKFIRE	LFESSTNNNK	DTKQKAGKLS	FISVGNKFKT
Rhinolophus ferrumequinum	AKLVGILDIL	DEENRLPQPS	DQHFTSAVHQ	KHKDHFRLTI	PRKSKLAVHR	NIRDDEGFIV	RHFAGAVCYE	TTQFVEKNND	ALHMSLESLI	CESRDKFIRE	LFESSTNNNK	DTKQKAGKLS	FISVGNKFKT
Rhinolophus pusillus	AKLVGILDIL	DEENRLPQPS	DQHFTSAVHQ	KHKDHFRLTI	PRKSKLAIHR	NIRDDEGFIV	RHFAGAVCYE	TTQFVEKNND	ALHMSLESLI	CESRDKFIRE	LFESSTNNNK	DTKQKAGKLS	FISVGNKFKT
Hipposideros pratti	AKLVGILDIL	DEENRLPQPS	DQHFTSAVHQ	KHRDHFRLTI	PRKSKLAVHR	NIRDDEGFII	RHFAGAVCYE	TTQFVEKNND	ALHMSLESLI	CESRDKFIRE	LFESSTNNNK	DTKQKAGKLS	FISVGNKFKT
Hipposideros armiger	AKLVGILDIL	DEENRLPQPS	DQHFTSAVHQ	KHRDHFRLTI	PRKSKLAVHR	NIRDDEGFII	RHFAGAVCYE	TTQFVEKNND	ALHMSLESLI	CESRDKFIRE	LFESSTNNNK	DTKQKAGKLS	FISVGNKFKT
Megaderma lyra	AKLVGILDIL	DEENRLPQPS	DQHFTSAVHQ	KHKDHFRLTI	PRKSKLAVHR	NIRDDEGFII	RHFAGAVCYE	TTQFVEKNND	ALHMSLESLI	CESRDKFIRE	LFESSTNNNK	DTKQKAGKLS	FISVGNKFKT
Myotis ricketti	AKLVGILDIL	DEENRLPQPS	DQHFTSAVHQ	KHKDHFRLTI	PRKSKLAIHR	NIRDDEGFVI	RHFAGAVCYE	TTQFVEKNND	ALHMSLESLI	CESRDKFIRE	LFETSTNNNK	DTKQKAGKLG	FISVGNKFKT
Pipistrellus abramus	AKLVGILDIL	DEENRLPQPS	DQHFTSAVHQ	KHKDHFRLTI	PRKSKLAIHR	NIRDDEGFII	RHFAGAVCYE	TTQFVEKNND	ALHMSLESLI	CESRDKFIRD	LFETSTNNNK	DTKQKAGKLG	FISVGNKFKT
Tadarida plicata	AKLVGILDIL	DEENRLPQPS	DQHFTSAVHQ	KHKDHFRLTI	PRKSKLAVHR	NIRDDEGFII	RHFAGAVCYE	TTQFVEKNND	ALHMSLESLI	CESRDKFIRE	LFESSTNNNK	DTKQKAGKLS	FISVGNKFKT
Pteronotus parnellii	AKLVGILDIL	DEENRLPQPS	DQHFTSAVHQ	KHKDHFRLTI	PRKSKLAVHR	NIRDDEGFIV	RHFAGAVCYE	TTQFVEKNND	ALHMSLESLI	CESRDKFIRE	LFESSTNNNK	DTKQKAGKLS	FISVGNKFKT
Mormoops megalophylla	AKLVGILDIL	DEENRLPQPS	DQHFTSAVHQ	KHKDHFRLTI	PRKSKLAVHR	NIRDDEGFII	RHFAGAVCYE	TTQFVEKNND	ALHMSLESLI	CESRDKFIRE	LFESSTNNNK	DTKQKAGKLS	FISVGNKFKT
Artibeus lituratus	AKLVGILDIL	DEENRLPQPS	DQHFTSAVHQ	KHKDHFRLTI	PRKSKLAVHR	NIRDDEGFIV	RHFAGAVCYE	TTQFVEKNND	ALHMSLESLI	CESRDKFIRE	LFESSTNNNK	DTKQKAGKLS	FISVGNKFKT
Leptonycteris yerbabuenae	AKLVGILDIL	DEENRLPQPS	DQHFTSAVHQ	KHKDHFRLTI	PRKSKLAVHR	NIRDDEGFIV	RHFAGAVCYE	TTQFVEKNND	ALHMSLESLI	CESRDKFIRE	LFESSTNNNK	DTKQKAGKLS	FISVGNKFKT

			**										
	6666666666	6666666666	6666666666	6666666666	6666666667	7777777777	7777777777	7777777777	7777777777	7777777777	7777777777	7777777777	7777777777
	5555555556	6666666667	7777777778	8888888889	9999999990	0000000001	1111111112	2222222223	3333333334	4444444445	5555555556	6666666667	7777777778
	1234567890	1234567890	1234567890	1234567890	1234567890	1234567890	1234567890	1234567890	1234567890	1234567890	1234567890	1234567890	1234567890
Human	QLNLLLDKLR	STGASFIRCI	KPNLKMTSHH	FEGAQILSQL	QCSGMVSVLD	LMQGGYPSRA	SFHELYNMYK	KYMPDKLARL	DPRLFCKALF	KALGLNENDY	KFGLTKVFFR	PGKFAEFDQI	MKSDPDHLAE
Chimpanzee	QLNLLLDKLR	STGASFIRCI	KPNLKMTSHH	FEGAQILSQL	QCSGMVSVLD	LMQGGYPSRA	SFHELYNMYK	KYMPDKLARL	DPRLFCKALF	KALGLNENDY	KFGLTKVFFR	PGKFAEFDQI	MKSDPDHLAE
Panda	QLNLLLDKLR	STGASFIRCI	KPNLKMTSHH	FEGAQILSQL	QCSGMVSVLD	LMQGGFPSRA	SFHELYNMYK	KYMPDKLARL	DPRLFCKALF	KALGLNEIDY	KFGLTKVFFR	PGKFAEFDQI	MKSDPDHLAK
Mouse	QLNLLLDKLR	STGASFIRCI	KPNLKMTSHH	FEGAQILSQL	QCSGMVSVLD	LMQGGFPSRA	SFHELYNMYK	KYMPEKLARL	DPRLFCKALF	KALGLNEVDY	KFGLTKVFFR	PGKFAEFDQI	MKSDPDHLAE
Rat	QLNLLLDKLR	STGASFIRCI	KPNLKMTSHH	FEGAQILSQL	QCSGMVSVLD	LMQGGFPSRA	SFHELYNMYK	KYMPEKLARL	DPRLFCKALF	KALGLNEVDY	KFGLTKVFFR	PGKFAEFDQI	MKSDPDHLAE
Cow	QLNLLLDKLR	STGASFIRCI	KPNLKMTSHD	FEGAQILSQL	QCSGMVSVLD	LMQGGFPSRA	SFHELYNMYK	KYMPDKLARL	DPRLFCKALF	KALGLNEVDY	KFGLTKVFFR	PGKFAEFDQI	MKSDPDHLAQ
Dog	QLNLLLDKLR	STGASFIRCI	KPNLKMTSHH	FEGAQILSQL	QCSGMVSVLD	LMQGGFPSRA	SFHELYNMYK	KYMPDKLARL	DPRLFCKALF	KALGLNEIDY	KFGLTKVFFR	PGKFAEFDQI	MKSDPDHLAE
Horse	QLNLLLDKLR	STGASFIRCI	KPNLKMTSHD	FEGAQILSQL	QCSGMVSVLD	LMQGGFPSRA	SFHELYNMYK	KYMPDKLARL	DPRLFCKALF	KALGLNEIDY	KFGLTKVFFR	PGKFAEFDQI	MKSDPDHLAK
Pig	QLNLLLDKLR	STGASFIRCI	KPNLKMTSHH	FEGAQILSQL	QCSGMVSVLD	LMQGGFPSRA	SFHEVYNMYK	KSLPDKLARL	DPRLFCKALF	KALGLNEIDY	KFGLTKVFFR	PGKFAEFDQI	MKSDPDHLAE
Cynopterus sphinx	QLNLLLDKLR	STGASFIRCI	KPNLKMVGHH	FEGAQILSQL	QCSGMVSVLD	LMQGGFPSRA	SFHELYNMYK	KYMPEKLARL	DPRLFCKALF	KALGLNEIDY	KFGLTKVFFR	PGKFAEFDQI	MKSDPDHLAE
Rousettus leschenaultii	QLNLLLDKLR	STGASFIRCI	KPNLKMVGHQ	FEGAQILSQL	QCSGMVSVLD	LMQGGFPSRA	SFHELYNMYK	KYMPDKLARL	DPRLFCKALF	KALGLNEVDY	KFGLTKVFFR	PGKFAEFDQI	MKSDPDHLAE
Eonycteris spelaea	QLNLLLDKLR	STGASFIRCI	KPNLKMVGHH	FEGAQILSQL	QCSGMVSVLD	LMQGGFPSRA	SFHELYNMYK	KYMPDKLARL	DPRLFCKALF	KALGLNEIDY	KFGLTKVFFR	PGKFAEFDQI	MKSDPDHLAE
Rhinolophus ferrumequinum	QLNLLLDKLR	STGASFIRCI	KPNLKMTSHD	FEGAQILSQL	QCSGMVSVLD	LMQGGFPSRA	SFHELYNMYK	KYMPDKLARL	DPRLFCKALF	KALGLNEIDY	KFGLTKVFFR	PGKFAEFDQI	MKSDPDHLAE
Rhinolophus pusillus	QLNLLLDKLR	STGASFIRCI	KPNLKMTSHD	FEGAQILSQL	QCSGMVSVLD	LMQGGFPSRA	SFHELYNMYK	KYMPDKLARL	DPRLFCKALF	KALGLNEIDY	KFGLTKVFFR	PGKFAEFDQI	MKSDPDHLAE
Hipposideros pratti	QLNLLLDKLR	STGASFIRCI	KPNLKMTSHD	FEGAQILSQL	QCSGMVSVLD	LMQGGFPSRA	SFHELYNMYK	KYMPDKLARL	DPRLFCKALF	KALGLNEIDY	KFGLTKVFFR	PGKFAEFDQI	MKSDPDHLAE
Hipposideros armiger	QLNLLLDKLR	STGASFIRCI	KPNLKMTSHD	FEGAQILSQL	QCSGMVSVLD	LMQGGFPSRA	SFHELYNMYK	KYMPDKLARL	DPRLFCKALF	KALGLNEIDY	KFGLTKVFFR	PGKFAEFDQI	MKSDPDHLAE
Megaderma lyra	QLNLLLDKLR	STGASFIRCI	KPNLKMTSHH	FEGAQILSQL	QCSGMVSVLD	LMQGGFPSRA	SFHELYNMYK	KYMPDKLARL	DPRLFCKALF	KALGLNEIDY	KFGLTKVFFR	PGKFAEFDQI	MKSDPDHLAE
Myotis ricketti	QLNLLLDKLR	STGASFIRCI	KPNLKMTSHD	FEGAQILSQL	QCSGMVSVLD	LMQGGFPSRA	SFHELYNMYK	KYMPDKLARL	DPRLFCKALF	KALGLNEIDY	KFGLTKVFFR	PGKFAEFDQI	MKSDPDHLAE
Pipistrellus abramus	QLNLLLDKLR	STGASFIRCI	KPNLKMTSHD	FEGAQILSQL	QCSGMVSVLD	LMQGGFPSRA	SFHELYNMYK	KYMPDKLARL	DPRLFCKALF	KALGLNEIDY	KFGLTKVFFR	PGKFAEFDQI	MKSDPDHLAE
Tadarida plicata	QLNLLLDKLR	STGASFIRCI	KPNLKMTSHD	FEGAQILSQL	QCSGMVSVLD	LMQGGFPSRA	SFHELYNMYK	KYMPDKLARL	DPRLFCKALF	KALGLNEIDY	KFGLTKVFFR	PGKFAEFDQI	MKSDPDHLAE
Pteronotus parnellii	QLNLLLDKLR	STGASFIRCI	KPNLKMTSHH	FEGAQILSQL	QCSGMVSVLD	LMQGGFPSRA	SFHELYNMYK	KYMPDKLARL	DPRLFCKALF	KALGLNEVDY	KFGLTKVFFR	PGKFAEFDQI	MKSDPDHLAE
Mormoops megalophylla	QLNLLLDKLR	STGASFIRCI	KPNLKMTSHH	FEGAQILSQL	QCSGMVSVLD	LMQGGFPSRA	SFHELYNMYK	KYMPDKLARL	DPRLFCKALF	KALGLNEIDY	KFGLTKVFFR	PGKFAEFDQI	MKSDPDHLAE
Artibeus lituratus	QLNLLLDKLR	STGASFIRCI	KPNLKMTSHH	FEGAQILSQL	QCSGMVSVLD	LMQGGFPSRA	SFHELYNMYK	KYMPDKLARL	DPRLFCKALF	KALGLNEIDY	KFGLTKVFFR	PGKFAEFDQI	MKSDPDHLAE
Leptonycteris yerbabuenae	QLNLLLDKLR	STGASFIRCI	KPNLKMTSHH	FEGAQILSQL	QCSGMVSVLD	LMQGGFPSRA	SFHELYNMYK	KYMPDKLARL	DPRLFCKALF	KALGLNEIDY	KFGLTKVFFR	PGKFAEFDQI	MKSDPDHLAE

```
                                       *                                                                                                                          *
                            7777777777 7777777778 8888888888 8888888888 8888888888 8888888888 8888888888 8888888888 8888888888 8888888888 8888888888 8888888889 9999999999
                            8888888889 9999999990 0000000001 1111111112 2222222223 3333333334 4444444445 5555555556 6666666667 7777777778 8888888889 9999999990 0000000001
                            1234567890 1234567890 1234567890 1234567890 1234567890 1234567890 1234567890 1234567890 1234567890 1234567890 1234567890 1234567890 1234567890
Human                       LVKRVNHWLT CSRWKKVQWC SLSVIKLKNK IKYRAEACIK MQKTIRMWLC KRRHKPRIDG LVKVGTLKKR LDKFNEVVSV LKDGKPEMNK QIKNLEISID TLMAKIKSTM MTQEQIQKEY DALVKSSEEL
Chimpanzee                  LVKRVNHWLT CSRWKKVQWC SLSVIKLKNK IKYRAEACIK MQKTIRMWLC KRRHKPRIDG LVKVGTLKKR LDKFNEVVSV LKDGKPEMNK QIKDLEISID ALMAKIKSTM MTQEQIQKEY DALVKSSEEL
Panda                       LVKRVNHWLI CSRWKKVQWC SLSVIKLKNK IKYRAEACIK MQKTVRMWLC KRRHKPRIDG LVKVGTLKKR LGKFNEVVST LKDGKPEVNK QVKDLEISID ALMAKIKSTM MTREQIQREY DALVKSSEEL
Mouse                       LVKRVNLWLV CSRWKKVQWC SLSVIKLKNK IKYRAEACIK MQKTIRMWLC KRRHKPRIDG LVKVGTLKKR LDKFNEVVSA LKDGKPEVNR QIKNLEISID ALMAKIKSTM MTREQIQKEY DALVKSSEDL
Rat                         LVKRVNLWLV CSRWKKVQWC SLSVIKLKNK IKYRAEACIK MQKTIRMWLC KRRHKPRIDG LVKVGTLKKR LDKFNEVVSA LKDGKPEVNR QIKDLEISID ALMAKIKSTM MTREQIQKEY DALVKSSEDL
Cow                         LVKRVNHWLI CSRWKKVQWC SLSVIKLKNK IKYRAEACIK MQKTIRMWLC KRRHKPRIDG LVKVGTLKKR LDKFNEVVSA LKDGKAEMNK QVKDLEISID ALMAKIKSTM MTREQIQKEY DALVKSSEVL
Dog                         LVKRVNHWLI CSRWKKVQWC SLSVIKLKNK IKYRAEACIK MQKTIRMWLC KRRHKPRIDG LVKVGTLKKR LDKFNEVVSA LKDGKLEMNK QVKDLEISID ALMAKIKSTM MTREQIRREY DALVKSSEEL
Horse                       LVKRVNHWLV CSRWKKVQWC SLSVIKLKNK IKYRAEACIK MQKTVRMWLC KRRHQPRIDG LVKVGTLKKR LDRFNEVVSA LKDGKPEVNK QVKDLEVSID ALMAKIKSTM MTREQIQKEY DALVKSSEEL
Pig                         LVKRVNHWLI CSRWKKVQWC SLSVIKLKNK IKYRAEACIK MQKTIRMWLC KRRHKPRIDG LVKVGTLKKR LDKFNEVVSA LKDGKQEMSK QVKDLEISID ALMAKIKSTM MTREQIQKEY DALVKSSAVL
Cynopterus sphinx           LVKRVNHWLI YSRWKKVQWC SLSVIKLKNK IKYRAEACIK MQKTIRMWLC KRRHKPRIDG LVKVGTLKKR LDKFNEVVSA LKDGKPAVNK QVKDLEISID ALMAKIKSTM MTREQIQKEY DAVVKSSENL
Rousettus leschenaultii     LVRRVNHWLI YSRWKKVQWC SLSVIKLKNK IKYRAEACIK MQKTIRMWLC KRRHKPRIDG LVKVGTLKKR LDKFNEVVSA LKDGKPEVNK QVKDLEISID ALMAKIKSTM MTREQIQKEY DAVVKSSENL
Eonycteris spelaea          LVRRVNHWLI YSRWKKVQWC SLSVIKLKNK IKYRAEACIK MQKTIRMWLC KRRHKPRIDG LVKVGTLKKR LDKFNEVVSA LKDGKPEVNK QVKDLEISID ALMAKIKSTM MTREQIQKEY DAVVKSSENL
Rhinolophus ferrumequinum   LVKRVNHWLI CSRWKKVQWC SLSVIKLKNK IKYRAEACIK MQKTIRMWLC KRRHKPRIDG LVKVGTLKKR LDKFNEVVSA LKDGKPEVNK QVKDLEISID ALMAKIKSTM MTREQIQKEY DALVRSSEDL
Rhinolophus pusillus        LVKRVNHWLI CSRWKKVQWC SLSVIKLKNK IKYRAEACIK MQKTIRMWLC KRRHKPRIDG LVKVGTLKKR LDKFNEVVSA LKDGKPEVNK QVKDLEISID ALMAKIKSTM MTREQIQKEY DALVKSSEDL
Hipposideros pratti         LVKRVNHWLI CSRWKKVQWC SLSVIKLKNK IKYRAEACIK MQKTIRMWLC KRRHKPRIDG LVKVGTLKKR LDKFNEVVSA LKDGKPEVNK QVKDLEVSID ALMAKIKSTM MTREQIQKEY DALVKSSEEL
Hipposideros armiger        LVKRVNHWLI CSRWKKVQWC SLSVIKLKNK IKYRAEACIK MQKTIRMWLC KRRHKPRIDG LVKVGTLKKR LDKFNEVVSA LKDGKPEVNK QVKDLEVSID ALMAKIKSTM MTREQIQKEY DALVKSSEEL
Megaderma lyra              LVKRVNHWLI CSRWKKVQWC SLSVIKLKNK IKYRAEACIK MQKTIRMWLC KRRHKPRIDG LVKVGTLRKR IDKFNEVVSA LKDGKPEVNK QIKDLEISID ALMAKIKSTM MTREQIQKEY DALVKSSEEL
Myotis ricketti             LVKRVNHWLI CSRWKKVQWC SLSVIKLKNK IKYRAEACIK MQKTIRMWLC KRRHKPRIDG LVKVGTLKKR LDKFNEVVSA LKDGKPEVNK QVKDLEISID ALMAKIKSTM MTREQIQKEY DALVKSSEEL
Pipistrellus abramus        LVKRVNHWLI CSRWKKVQWC SLSVIKLKNK IKYRAEACIK MQKTIRMWLC KRRHKPRIDG LVKVGTLKKR LDKFNEVVSA LKDGKPEVNK QVKDLEVSID ALMAKIKSTM MTREQIQKEY DALVKSSEEL
Tadarida plicata            LVKRVNHWLI CSRWKKVQWC SLSVIKLKNK IKYRAEACIK MQKTIRMWLC KRRHKPRIDG LVKVGTLKKR LDKFNEVVSA LKDGKPEVNK QVQDLEVSID ALMAKIKSTM MTREQIQKEY DALVKSSEEL
Pteronotus parnellii        LVKRVNHWLI CSRWKKVQWC SLSVIKLKNK IKYRAEACIK MQKTIRMWLC KRRHKPRIDG LVKVGTLKKR LDKFNEVVSA LKEGKPEMNK QVKDLEISID ALMAKIKSTM MTREQIQQEY DALVKSSEEL
Mormoops megalophylla       LVKRVNHWLI CSRWKKVQWC SLSVIKLKNK IKYRAEACIK MQKTIRMWLC KRRHKPRIDG LVKVGTLKKR LDKFNEVVSA LKEGKPEMNK QVKDLEISID ALMAKIKSTM MTREQIQKEY DALVKSSEEL
Artibeus lituratus          LVKRVNHWLI CSRWKKIQWC SLSVIKLKNK IKYRAEACIK MQKTIRMWLC KRRHKPRIDG LVKVGTLKKR LDKFNEVVSA LKEGKPEMSK QVKDLEISID ALMAKIKSTM MTREQIQKEY DALVKSSEAL
Leptonycteris yerbabuenae   LVKRVNHWLI CSRWKKVQWC SLSVIKLKNK IKYRAEACIK MQKTIRMWLC KRRHKPRIDG LVKVGTLKKR LDKFNEVVSA LKEGKPEMSK QVKDLEISID ALMAKIKSTM MTRQQIQKEY DALVKSSEEL

                              *
                                                                                                                 1 1111111111 1111111111 1111111111 1111111111
                            9999999999 9999999999 9999999999 9999999999 9999999999 9999999999 9999999999 9999999999 9999999990 0000000000 0000000000 0000000000 0000000000
                            1111111112 2222222223 3333333334 4444444445 5555555556 6666666667 7777777778 8888888889 9999999990 0000000001 1111111112 2222222223 3333333334
                            1234567890 1234567890 1234567890 1234567890 1234567890 1234567890 1234567890 1234567890 1234567890 1234567890 1234567890 1234567890 1234567890
Human                       LSALQKKKQQ EEEAERLRRI QEEMEKERKR REEDEKRRRK EEEERRMKLE MEAKRKQEEE ERKKREDDEK RIQAEVEAQL ARQKEEESQQ QAVLEQERRD RELALRIAQS EAELISDEAQ AD-LALRRND
Chimpanzee                  LSALQKKKQQ EEEAERLRRI QEEMEKERKR REEDEKRRRK EEEERRMKLE MEAKRKQEEE ERKKREDDEK RIQAEVEAQL ARQREEESQQ QAVLEQERRD RELALRIAQS EAELISDEAQ AD-LALRRND
Panda                       LSALQRKKQQ EEEAERLRRI QEEMEKERKR REEDEQRRRK EEEERRMKLE MEAKRKQEEE ERKKREDDEK RIQAEVEKQL ARQREEESQQ QVVLEQERRD RELALRIAQS EAELISEEAQ AD-LSLRRID
Mouse                       LSALQKKKQQ EEEAERLRRI QEEMEKERKR REEDEERRRK EEEERRMKLE MEAKRKQEEE ERKKREDDEK RIQAEVEAQL ARQREEESQQ QAVLAQECRD RELALRIAQN ESELISDEAQ GD-MALRR--
Rat                         LSALQKKKQQ EEEAERLRRI QEEMEKERKR REEDEQRRRK EEEERRMKLE MEVKRKQEEE ERKKREDDEK RIQAEVEAQL ARQREEESQQ QAVLAQECRD RELALRIAQN ESELISDEAQ GD-TALRR--
Cow                         LSALQKKKQQ EEEAERLRRI QEEMEKERKR REEDEQRRRK EEEERRMKLE MEAKRKQEEE ERKKREDDEK RIQAEVEAQL ARQREEESQQ QAVLEQERRD RELALRIARS EAELIIDEAQ ADPAALRRIN
Dog                         LSALQKKKQQ EEEAERLKRI QEEMEKERKR REEDERRRRK EEEERRMKLE MEAKRKQEEE ERKKREDDEK RIQAEVEEQL ARQREEESQQ QAVLEQERRD RELALRIAQS EAELITDEAQ GD-LALRRIV
Horse                       LSALQRKKQQ EEEAERLRRI QEEMEKERKR REEDEQRRRK EEEERRMKLE MEAKRKQEEE ERKKREDDEK RIQAEVEEQL AQQREEESQQ QAVLEQERRD RELALRIAQS EAELISDEAQ AD-PALRSID
Pig                         LSALQKKKQQ EEEAERLRRI QEEMEKERKR REEDEQRRRK EEEERRMKLE MEAKRKQEEE ERKKREDDEK RIQAEVEAQL ARQREEESQQ QAVLEQERRD RELALRIAQS EAELISDEAQ AD-PGLRR--
Cynopterus sphinx           LSELQKKKQQ EEEAERLRRI QEEMEKERKR REEDEQRRRK EEEERQMKLE MEAKRKQEEE ERKKREDDEK RIQAEVEEQL ARQREEESQQ QAVLEQERRD RELALRIAQS EAELISDEAQ AD-LALRR--
Rousettus leschenaultii     LSGLQKKKQQ EEEAERLRRI QEEMEKERKR REEDEQRRRK EEEERRMKLE MEAKRKQEEE ERKKREDDEK RIQAEVEEQL ARQREEESQQ QAVLEQERRD RELALRIAQS EAELISDEAQ AD-LALRR--
Eonycteris spelaea          LSGLQKKKQQ EEEAERLRRI QEEMEKERKR REEDEQRRRR EEEERRMKLE MEAKRKQEEE ERKKREDDEK RIQAEVEEQL ARQREEESQQ QAVLEQERRD RELALRIAQS EAELISDEAQ AD-LALRR--
Rhinolophus ferrumequinum   LSALQKKKQQ EEEAERLRRI QEEMEKERKR REEDEQRRRK EEEERRMKLE MEAKRKQEEE ERKKREDDEK RIQAEVEEQL ARQREEESQQ QAVLEQERRD RELALRIAQS EAELISDEAQ AD-LALRR--
Rhinolophus pusillus        LSALQKKKQQ EEEAERLRRI QEEMEKERKR REEDEQRRRK EEEERRMKLE MEAKRKHEEE ERKKREDDEK RIQAEVEEQL ARQREEESQQ QAVLEQERRD RELALRIAQS EAELISDEAQ AD-LALRR--
Hipposideros pratti         LSALQKKKQQ EEEAERLRRI QEEMEKERKR REEDEQRRRK EEEERRMKLE MEAKRKQEEE ERKKREDDEK LIQAEVEEQL ARQREEESQQ QAVLEQERRD RELALRIAQS EAELISDEAQ AD-LALRR--
Hipposideros armiger        LSALQKKKQQ EEEAERLRRI QEEMEKERKR REEDEQRRRK EEEERRMKLE MEAKRKQEEE ERKKREDDEK LIQAEVEEQL ARQREEESQQ QAVLEQERRD RELALRIAQS EAELISDEAQ AD-LALRR--
Megaderma lyra              LSALQKKKQQ EEEAERLRRI QEEMEKERKR REEDEQRRRK EEEERRMKLD MEAKRKQEEE ERKKREDDEK RIQAEVEEQL ARQREEESQQ QAVLEQERRD RELALRIAQS EAELISDEAQ AD-LALRR--
Myotis ricketti             LSALQKKKQQ EEEAERLRRI QEEMEKERKR REEDEQRRRK EEEERRMKLE MEAKRKQEEE ERKKREDDEK RIQAEVEEQL ARQREEESQQ QAVLEQERRD RELALRIAQS EAELISDEAQ AD-LALRR--
Pipistrellus abramus        LSALQKKKQQ EEEAERLRRI QEEMEKERKR REEDEQRRRK EEEERRMKLE MEAKRKQEEE ERKKREDDEK RIQAEVEEQL ARQREEESQQ QAVLEQERRD RELALRIAQS EAELISDEAQ VD-LALRR--
Tadarida plicata            LSALQKKKQQ EEEAERLRRI QEEMEKERKR REEDEQRRRK EEEERRMKLE MEAKRKQEEE ERKKREDDEK RIQAEVEEQL ARQREEESQQ QAVLEQERRD RELALRIAQS EAELISDEAQ AD-LALRR--
Pteronotus parnellii        LSALQKKKQQ EEEAERLRRI QEEMEKERKR REEDEQRRRK EEEERRMKLE MEAKRKQEEE ERKKREDEEK RIQAEVEAQL ARQREEESQQ QAVLEQERRD RELALRIAQS EAELISDEAQ AD-LALRR--
Mormoops megalophylla       LSALQKKKQQ EEEAERLRRI QEEMEKERKR REEDEQRRRK EEEERRMKLE MEAKRKQEEE DRKKREDEEK RIQAEVEAQL ARQREEESQQ QAVLEQERRD RELALRIAQS EAELISDEAQ AD-LALRR--
Artibeus lituratus          LSALQKKKQQ EEEAERLRRI QEEMEKERKR REEDEQRRRK EEEERRMKLE MEAKRKQEEE ERKKREDEEK RIQAEVEEQL ARQREEESQQ QAVLEQERRD RELALRIAQS EAELISDEAQ AD-LALRR--
Leptonycteris yerbabuenae   LSALQKKKQQ EEEAERLRRI QEEMEKERKR REEDEQRRRK EEEERRMKLE MEAKRKQEEE ERKKREDEEK RIQAEVEAQL ARQREEESQQ QAVLEQERRD RELALRIAQS EAELISDEAQ AD-LALRR--

                                                                                                                                                                    *   *
                            1111111111 1111111111 1111111111 1111111111 1111111111 1111111111 1111111111 1111111111 1111111111 1111111111 1111111111 1111111111 1111111111
                            0000000000 0000000000 0000000000 0000000000 0000000000 0000000001 1111111111 1111111111 1111111111 1111111111 1111111111 1111111111 1111111111
                            4444444445 5555555556 6666666667 7777777778 8888888889 9999999990 0000000001 1111111112 2222222223 3333333334 4444444445 5555555556 6666666667
                            1234567890 1234567890 1234567890 1234567890 1234567890 1234567890 1234567890 1234567890 1234567890 1234567890 1234567890 1234567890 1234567890
Human                       GTRPKMTPEQ MAKEMSEFLS RGPAVLATKA AAGTKKYDLS KWKYAELRDT INTSCDIELL AACREEFHRR LKVYHAWKSK NKKRNTETEQ RAPKSVTDYD FAPFLNNSPQ QNPAAQIPAR QREIEMNRQQ
Chimpanzee                  GTRPKMTPEQ MAKEMSEFLS RGPAVQATKA AAGTKKYDLS KWKYAELRDT INTSCDIELL AACREEFHRR LKVYHAWKSK NKKRNTETEQ RAPKSVTDYD FAPFLNNSPQ QNPAAQIPAR QQEIEMNRQQ
Panda                       GARPKMTPEQ MEKEMSEFLS RGPAVQATKA AAGTKKYDLS KWKYAELRDT INTSCDIELL AACREEFHRR LKVYHAWKSK NKKRNTETEQ RAPKSVTDYD FAPFLNNSPQ QNPASQRPAR QQEIEMNRQQ
Mouse                       ---------- ---------- -GPAVQATKA AAGTKKHDLS KWKYAELRDT INTSCDIELL AACREEFHRR LKVYHAWKSK NKKRNTETEQ RAPKSVTDYD FAPFLNNSPQ QNPAAQLPAR QQEIDMKRQQ
Rat                         ---------- ---------- -GPAVQATKA AAGTKKHDLS KWKYAELRDT INTSCDIELL AACREEFHRR LKVYHAWKSK NKKRNTETEQ RAPKSVTDYD FAPFLNNSPQ QNPAAQLPAR QQEIEMNRQQ
Cow                         GTRRTMTPEQ MAKEMSEILS RGPAVQATKA AAGTKKHDLS KWKYAELRDT INTSCDIELL AACREEFHRR LKVYHAWKSK NKKRNTETEQ RAPKSVTDYD FAPFLNNSPQ QNPAAQLPAR QQEIEMNRQQ
Dog                         GARPKMTPEQ MEREMSEFLS RGPAVQATKA AAGTKKYDLS KWKYAELRDT INTSCDIELL AACREEFHRR LKVYHAWKSK NKKRNTETEQ RAPKSVTDYD FAPFLNNSPQ QNPASQLPAR QQEMEMNRQQ
Horse                       GRRPKMTPEQ MAKEISEFLS RSPAVQATKA AAGTKKHDLS KWKYAELRDT INTSCDIELL AACREEFHRR LKVYHAWKSK NKKRNTETEQ RAPKSVTDYD FAPFLNNSPQ QNPAVQLPAR QQEPERHRQQ
Pig                         ---------- ---------- -GPAVQATKA AAGTKKYDLS KWKYAELRDT INTSCDIELL AACREEFHRR LKVYHAWKSK NKKRNTETEQ RAPKSVTDY- --------AQ QNPAVQLPAR QQEIEMNRQQ
Cynopterus sphinx           ---------- ---------- -GPAVQATKA AAGTKKHDLS KWKYAELRDT INTSCDIELL AACREEFHRR LKVYHAWKSK NKKRNTETEQ RAPKSVTDYD FAVFLNNSPQ QNPAAQLPAR QQEIIMNRKQ
Rousettus leschenaultii     ---------- ---------- -GPAVQATKA AAGTKKHDLS KWKYAELRDT INTSCDIELL AACREEFHRR LKVYHAWKSK NKKRNTETEQ RAPKSVTDYD FAVFLNNSPQ QNPAAQLPAR QQEIIMNRKQ
Eonycteris spelaea          ---------- ---------- -GPAVQATKA AAGSKKHDLS KWKYAELRDT INTSCDIELL AACREEFHRR LKVYHAWKSK NKKRNTETEQ RAPKSVTDYD FAVFLNNSPQ QNPAAQLPAR QQEIIMNRKQ
Rhinolophus ferrumequinum   ---------- ---------- -GPAVQATKA AAGTKKHDLS KWKYAELRDT INTSCDIELL AACREEFHRR LKVYHAWKSK NKKRNTETEQ RAPKSVTDYD FAPFLNNSPQ QNPAAQLPAR QQEIEMNRQQ
Rhinolophus pusillus        ---------- ---------- -GPAVQATKA AAGTKKHDLS KWKYAELRDT INTSCDIELL AACREEFHRR LKVYHAWKSK NKKRNTETEQ RAPKSVTDYD FAPFLNNSPQ QNPAAQLPAR QQEIEMNRQQ
Hipposideros pratti         ---------- ---------- -GPAVQATKA AAGTKKHDLS KWKYAELRDT INTSCDIELL AACREEFHRR LKVYHAWKSK NKKRNTETEQ RAPKSVTDYD FAPFLNNSPQ QNPAAQLPAR QQEMEMNRQQ
Hipposideros armiger        ---------- ---------- -GPAVQATKA AAGTKKHDLS KWKYAELRDT INTSCDIELL AACREEFHRR LKVYHAWKSK NKKRNTETEQ RAPKSVTDYD FAPFLNNSPQ QNPAAQLPAR QQEIEMNRQQ
Megaderma lyra              ---------- ---------- -GPAVQATKA AAGTKKHDLS KWKYAELRDT INTSCDIELL AACREEFHRR LKVYHAWKSK NKKRNTETEQ RAPKSVTDYD FAPFLNSSPQ QNPAAQRPAR QQAVETDRQQ
Myotis ricketti             ---------- ---------- -GPAVQATKA AAGTKKHDLS KWKYAELRDT INTSCDIELL AACREEFHRR LKVYHAWKSK NKKRNTETEQ RAPKSVTDYD FAPFLNNSPQ QNPAAQLPAR QQEIEMNRQQ
Pipistrellus abramus        ---------- ---------- -GPAVQATKA AAGTKKHDLS KWKYAELRDT INTSCDIELL AACREEFHRR LKVYHAWKSK NKKRNTETEQ RAPKSVTDYD FATFLNNSPQ QNPAAQLPAR QQEIEMNRQQ
Tadarida plicata            ---------- ---------- -GPAVQATKA AAGTKKHDLS KWKYAELRDT INTSCDIELL AACREEFHRR LKVYHAWKSK NKKRNTETEQ RAPKSVTDYD FAPFLNNSPQ QNPAAQLPAR QQEIEMNRQQ
Pteronotus parnellii        ---------- ---------- -GPAVQATKA AAGTKKYDLS KWKYAELRDT INTSCDIELL AACREEFHRR LKVYHAWKSK NKKRNTETEQ RAPKSVTDYD FAPFLNNSPQ QNPAAQLPAR QQQIEMNRQQ
Mormoops megalophylla       ---------- ---------- -GPAVQATKA AAGTKKYDLS KWKYAELRDT INTSCDIELL AACREEFHRR LKVYHAWKSK NKKRNTETEQ RAPKSVTDYD FAPFLNNSPQ QNPAVQLPAR QQQIEMNRQQ
Artibeus lituratus          ---------- ---------- -GPAVQATKA AAGTKKYDLS KWKYAELRDT INTSCDIELL AACREEFHRR LKVYHAWKSK NKKRNTETEQ RAPKSVTDYD FAPFLNNSPQ QNPAAQLPAR QQQIEMNRQQ
Leptonycteris yerbabuenae   ---------- ---------- -GPAVQATKA AAGSKKYDLS KWKYAELRDT INTSCDIELL AACREEFHRR LKVYHAWKSK NKKRNTEAEQ RAPKSVTDYD FAPFLNNSPQ QNPAAQLPAR QQQIEMNRQQ
```

	1111111111	1111111111	1111111111	1111111111	1111111111	1111111111	1111111111	1111111111	1111111111	1111111111	1111111111	1111111
	1111111111	1111111111	1111111112	2222222222	2222222222	2222222222	2222222222	2222222222	2222222222	2222222222	2222222222	2222222
	7777777778	8888888889	9999999990	0000000001	1111111112	2222222223	3333333334	4444444445	5555555556	6666666667	7777777778	8888888
	1234567890	1234567890	1234567890	1234567890	1234567890	1234567890	1234567890	1234567890	1234567890	1234567890	1234567890	1234567
Human	RFFRIPFIRP	ADQYKDPQSK	KKGWWYAHFD	GPWIARQMEL	HPDKPPILLV	AGKDDMEMCE	LNLEETGLTR	KRGAEILPRQ	FEEIWERCGG	IQYLQNAIES	RQARPTYATA	MLQSLLK
Chimpanzee	RFFRIPFIRP	ADQYKDPQSK	KKGWWYAHFD	GPWIARQMEL	HPDKPPILLV	AGKDDMEMCE	LNLEETGLTR	KRGAEILPRQ	FEEIWERCGG	IQYLQNAIES	RQARPTYATA	MLQSLLK
Panda	RFFRIPFIRP	ADQYKNPQNK	KKGWWYAHFD	GPWIARQMEL	HPDKPPILLV	AGKDDMEMCE	LNLEETGLTR	KRGAEILPRQ	FEEIWERCGG	IQYLQKAIES	RQARPTYATA	MLQNLFK
Mouse	RFFRIPFIRP	ADQYKDPQNK	KKGWWYAHFD	GPWIARQMEL	HPDKPPILLV	AGKDDMEMCE	LNLEETGLTR	KRGAEILPRQ	FEEIWERCGG	IQYLQSAIES	RQARPTYATA	MLQNLLK
Rat	RFFRIPFIRP	ADQYKDPQNK	KKGWWYAHFD	GPWIARQMEL	HPDKPPILLV	AGKDDMEMCE	LNLEETGLTR	KRGAEILPRQ	FEEIWERCGG	IQYLQSAIES	RQARPTYATA	MLQNLLK
Cow	RFFRIPFIRP	ADQYKDPQNK	KKGWWYAHFD	GPWIARQMEL	HPDKPPILLV	AGKDDMEMCE	LNLEETGLTR	KRGAEILPRQ	FEEIWERCGG	IQYLQSAIES	RQARPTYATA	MLQNLLK
Dog	RFFRIPFIRP	ADQYKDPQNK	KKGWWYAHFD	GPWIARQMEL	HPDKPPILLV	AGKDDMEMCE	LNLEETGLTR	KRGAEILPRQ	FEEIWERCGG	IQYLQNAIES	RQARPTYATA	MLQNLLK
Horse	RFFRIPFIRP	ADQYKDPQNK	KKGWWYAHFD	GPWIARQMEL	HPDKPPILLV	AGKDDMEMCE	LNLEETGLTR	KRGAEILPRQ	FEEVWERCGG	IQYLQSAIES	RQARPTHATA	MLQSLLQ
Pig	RFFRIPFIRS	ADQYKDPQNK	KKGWWYAHFD	GPWIARQMEL	HPDKPPILLV	AGKDDMEMCE	LNLEETGLTR	KRGAEILPRQ	FEEIWERCGG	IQYLQNAIES	RQARPTYATA	MLQNLLK
Cynopterus sphinx	RFFRIPFIRP	ADQYKDPQNK	KKGWWYAHFD	GPWIARQMEL	HPDKPPILLV	AGKDDMEMCE	LNLEETGLTR	KRGAEILPRQ	FEEIWERCGG	IQYLQNAIES	RQARPTYATA	MLQSLLK
Rousettus leschenaultii	RFFRIPFIRP	ADQYKDPQNK	KKGWWYAHFD	GPWIARQMEL	HPDKPPILLV	AGKDDMEMCE	LNLEETGLPR	KRGAEILPRQ	FEEIWERCGG	IQYLQNAIES	RQARPTYATA	MLQSLLK
Eonycteris spelaea	RFFRIPFIRP	ADQYKDPQNK	KKGWWYAHFD	GPWIARQMEL	HPDKPPILLV	AGKDDMEMCE	LNLEETGLPR	KRGAEILPRQ	FEEIWERCGG	IQYLQNAIES	RQARPTYATA	MLQSLLK
Rhinolophus ferrumequinum	RFFRIPFIRP	ADQYKDPQNK	KKGWWYAHFD	GPWIARQMEL	HPDKPPILLV	AGKDDMEMCE	LNLEETGLTR	KRGAEILPRQ	FEEIWERCGG	IQYLQNAIES	RQARPTYATA	MLQSLLK
Rhinolophus pusillus	RFFRIPFIRP	ADQYKDPQNK	KKGWWYAHFD	GPWIARQMEL	HPDKPPILLV	AGKDDMEMCE	LNLEETGLTR	KRGAEILPRQ	FEEIWERCGG	IQYLQNAIES	RQARPTYATA	MLQSLLK
Hipposideros pratti	RFFRIPFIRP	ADQYKDPQNK	KKGWWYAHFD	GPWIARQMEL	HPDKPPILLV	AGKDDMEMCE	LNLEETGLTR	KRGAEILPRQ	FEEIWERCGG	IQYLQNAIQS	RQARPTYATA	MLQSLLK
Hipposideros armiger	RFFRIPFIRP	ADQYKDPQNK	KKGWWYAHFD	GPWIARQMEL	HPDKPPILLV	AGKDDMEMCE	LNLEETGLTR	KRGAEILPRQ	FEEIWERCGG	IQYLQNAIQS	RQARPTYATA	MLQSLLK
Megaderma lyra	RFFRIPFIRP	ADQYKDPQSK	KKGWWYAHFD	GPWIARQMEL	HPDKPPILLV	AGKDDMEMCE	LNLEETGLTR	KRGAEILPRQ	FEEIWERCGG	IQYLQNAIES	RQARPTYATA	MLQSLLK
Myotis ricketti	RFFRIPFIRP	ADQYKDPQNK	KKGWWYAHFD	GPWIARQMEL	HPDKPPILLV	AGKDDMEMCE	LNLEETGLTR	KRGAEILPRQ	FEEIWERCGG	IQYLQNAIES	RQARPTYATA	MLQSLLK
Pipistrellus abramus	RFFRIPFIRP	ADQYKDPQNK	KKGWWYAHFD	GPWIARQMEL	HPDKPPILLV	AGKDDMEMCE	LNLEETGLTR	KRGAEILPRQ	FEEIWERCGG	IQYLQKAIES	RQARPTYATA	MLQSLLK
Tadarida plicata	RFFRIPFIRP	ADQYKDPQNK	KKGWWYAHFD	GPWIARQMEL	HPDKPPILLV	AGKDDMEMCE	LNLEETGLTR	KRGAEILPRQ	FEEIWERCGG	IQYLQNAIES	RQARPTYATA	MLQSLLK
Pteronotus parnellii	RFFRIPFIRP	ADQYKDPQNK	KKGWWYAHFD	GPWIARQMEL	HPDKPPILLV	AGKDDMEMCE	LNLEETGLTR	KRGAEILPRQ	FEEIWERCGG	IQYLQNAIES	RQARPTYATA	MLQSLLK
Mormoops megalophylla	RFFRIPFIRP	ADQYKDPQNK	KKGWWYAHFD	GPWIARQMEL	HPDKPPILLV	AGKDDMEMCE	LNLEETGLTR	KRGAEILPRQ	FEEIWERCGG	IQYLQNAIES	RQARPTYATA	MLQSLLK
Artibeus lituratus	RFFRIPFIRP	ADQYKDPQNK	KKGWWYAHFD	GPWIARQMEL	HPDKPPILLV	AGKDDMEMCE	LNLEETGLTR	KRGAEILPRQ	FEEVWERCGG	IQYLQSAIES	RQARPTYATA	MLQSLLK
Leptonycteris yerbabuenae	RFFRIPFIRP	ADQYKDPQNK	KKGWWYAHFD	GPWIARQMEL	HPDKPPILLV	AGKDDMEMCE	LNLEETGLTR	KRGAEILPRQ	FEEIWERCGG	IQYLQNAIES	RQARPTYATA	MLQSLLK
