# Supplementary material for: Adaptive Evolution of the Myo6 Gene in Old World Fruit Bats (Family: Pteropodidae)
Source: PLoS One. 2013 Apr 19;8(4):e62307. doi: 10.1371/journal.pone.0062307 (PMC3631194; doi:10.1371/journal.pone.0062307)
Supplement: Table S1 — List of species analyzed in this study. (DOC) [file pone.0062307.s004.doc]

**Table S1. List of species analyzed in this study**

| **Order** | **Suborder** | — | — | **Species name** | **Family** | **Echolocating^a^** | **Food habits^b^** | **Accession number** |
| --- | --- | --- | --- | --- | --- | --- | --- | --- |
| Chiroptera | Yinpterochiroptera | Old World fruit bats | — | *Cynopterus sphinx* | Pteropodidae | Nonecholocating | F | JX023445 |
|  |  |  | — | *Rousettus leschenaultii* |  | Tongue-clicking | F | JX023457 |
|  |  |  | — | *Eonycteris spelaea* |  | Nonecholocating | N | JX023446 |
|  |  | Yinpterochiroptera echolocating bats | CF rhinolophoid bats | *Rhinolophus ferrumequinum* | Rhinolophidae | CF echolocation | I | JX023455 |
|  |  |  |  | *Rhinolophus* *pusillus* |  |  | I | JX023456 |
|  |  |  |  | *Hipposideros pratti* | Hipposideridae |  | I | JX023448 |
|  |  |  |  | *Hipposideros* *armiger* |  |  | I | JX023447 |
|  |  |  | — | *Megaderma lyra* | Megadermatidae | FM echolocation | C and I | JX023450 |
|  | Yangochiroptera | Yangochiroptera echolocating bats | — | *Mormoops megalophylla* | Mormoopidae | FM echolocation | I | JX023451 |
|  |  |  | — | *Pteronotus parnellii* |  | CF echolocation | I | JX023454 |
|  |  |  | New World fruit bats | *Artibeus lituratus* | Phyllostomidae | FM echolocation | F | JX023444 |
|  |  |  |  | *Leptonycteris yerbabuenae* |  | FM echolocation | N | JX023449 |
|  |  |  | — | *Myotis ricketti* | Vespertilionidae | FM echolocation | I and P | JX023452 |
|  |  |  | — | *Pipistrellus abramus* |  | FM echolocation | I | JX023453 |
|  |  |  | — | *Tadarida plicata* | Molossidae | FM echolocation | I | JX023458 |
| Primates | — | — | — | *Homo sapiens* | ­Hominidae | — | — | NM_004999 |
|  | — | — | — | *Pan troglodytes* |  | — | — | XM_001144940 |
| Rodentia | — | — | — | *Mus musculus* | Muridae | — | — | NM_001039546 |
|  | — | — | — | *Rattus norvegicus* |  | — | — | XM_001061392 |
| Artiodactyla | — | — | — | *Bos taurus* | Bovidae | — | — | NM_001206072 |
|  | — | — | — | *Sus scrofa* | Suidae | — | — | NM_214021 |
| Perissodactyla | — | — | — | *Equus caballus* | Equidae | — | — | XM_001503608 |
| Carnivora | — | — | — | *Canis familiaris* | Canidae | — | — | XM_862495 |
|  | — | — | — | *Ailuropoda melanoleuca* | Ursidae | — | — | XM_002923922 |

^a^CF: constant-frequency; FM: frequency-modulated;

^b^F: frugivorous; N: nectarivorous; I: insectivorous; C: carnivorous; P: piscivorous.
