# Supplementary material for: Adaptive Evolution of the Myo6 Gene in Old World Fruit Bats (Family: Pteropodidae)
Source: PLoS One. 2013 Apr 19;8(4):e62307. doi: 10.1371/journal.pone.0062307 (PMC3631194; doi:10.1371/journal.pone.0062307)
Supplement: Table S2 — Information of primers used for Myo6 coding sequences PCR and real-time PCR. (DOC) [file pone.0062307.s005.doc]

**Table S2. Information of primers used for *Myo6* coding sequences PCR and real-time PCR**

| **Primers for PCR amplification** | |
| --- | --- |
| **Fragment** | **Primers** |
| Fragment 1 | Forward primer: 5’-ATGGAGGACGGAAAGCCCGTTTG-3’  Reverse primer: 5’-TTTGGTGCCCCCTGCTGTTGTTAG-3’ |
| Fragment 2 | Forward primer: 5’-GGTTTGGATCAAGATGATCTTCGC-3’  Reverse primer: 5’-AAGACAGACACCATACCTGAACAC-3’ |
| Fragment 3 | Forward primer: 5’-GAAAACTTGGCTTCATCAGTGTGG-3’  Reverse primer: 5’-TGCTTCCATCTCAAGTTTCATCCG-3’ |
| Fragment 4 | Forward primer: 5’-AACAACGTCGAAGAAAGGAAGAGG-3’  Reverse primer: 5’-CTACTTTAACAGATTCTGCAGCAT-3’ |
| **Primers for Real-time PCR** | |
| **Gene** | **Primers^*^** |
| *Gapdh* (internal control) | Forward primer: 5’-ATGGGTGTGAACCAYGASAAGT-3’  Reverse primer: 5’-GGTCATGAGTCCYTCCACRAT-3’ |
| *Myo6* | Forward primer: 5’-CGCATCCCGTTCATCCGC-3’  Reverse primer: 5’-ATGTCATCCTTACCAGCCAC-3’ |

^*^Y = C+T, S = G+C, R = A+G.
